# Gut microbiome composition and functional potential associate with incident type 2 diabetes in 4,685 adults from a Swedish prospective cohort

**DOI:** 10.1016/j.xcrm.2026.102835

**Published:** 2026-05-27

**Authors:** Gaël Toubon, Fredrik Boulund, Cecilia Martinez Escobedo, Carl Brunius, Lars Engstrand, Susanna C. Larsson, Elise Nordin, Ina Schuppe-Koistinen, Alicja Wolk, Clemens Wittenbecher, Rikard Landberg

**Affiliations:** 1 Division of Food and Nutrition Science, Department of Life Sciences, Chalmers University of Technology, Gothenburg, Sweden; 2 Centre for Translational Microbiome Research, Department of Microbiology, Tumor and Cell Biology, Karolinska Institutet, Stockholm, Sweden; 3 Unit of Cardiovascular and Nutritional Epidemiology, Institute of Environmental Medicine, Karolinska Institutet, Stockholm, Sweden; 4 Medical Epidemiology, Department of Surgical Sciences, Uppsala University, Uppsala, Sweden

**Keywords:** gut microbiome, microbial functional potential, shotgun sequencing, prospective cohort study, machine learning, incident type 2 diabetes

## Abstract

Cross-sectional studies link gut microbiome alterations to type 2 diabetes (T2D), but prospective evidence remains limited. We aim to identify taxonomic and functional features associated with future T2D risk. We analyze shotgun metagenomic data from 4,685 participants (mean age, 73.9 years; 49.0% women) in the Swedish SIMPLER cohort, followed for a median 5.3 years, during which 383 developed T2D. Six species are associated with increased T2D risk: *Desulfovibrio piger*, *Alistipes communis*, *Alistipes finegoldii*, *Akkermansia muciniphila*, *Ruminococcus gnavus*, and GGB3614_SGB4886 (Lachnospiraceae), while three are protective: Erysipelotrichaceae bacterium, *Coprococcus catus*, and Clostridia unclassified SGB6317. We observe context-specific associations, including a dietary fiber-modified effect for *A. muciniphila* indicative of diet-dependent patterns. Three gut metabolic modules are associated with incident T2D: asparagine degradation (higher risk), mannose degradation, and the non-oxidative pentose phosphate pathway (lower risk). These prospective findings offer insights into T2D etiology and may support microbiome-informed strategies for risk prediction and prevention.

## Highlights

• Large-scale prospective metagenomics (*n* = 4,685) links gut microbiome to T2D risk
• Findings are derived from a T2D-free and antidiabetic medication-naive population
• Nine species and three gut metabolic modules associate with future risk of T2D
• Dietary fiber modifies the association between *A. muciniphila* and T2D risk

## Graphical abstract

**Figure:**
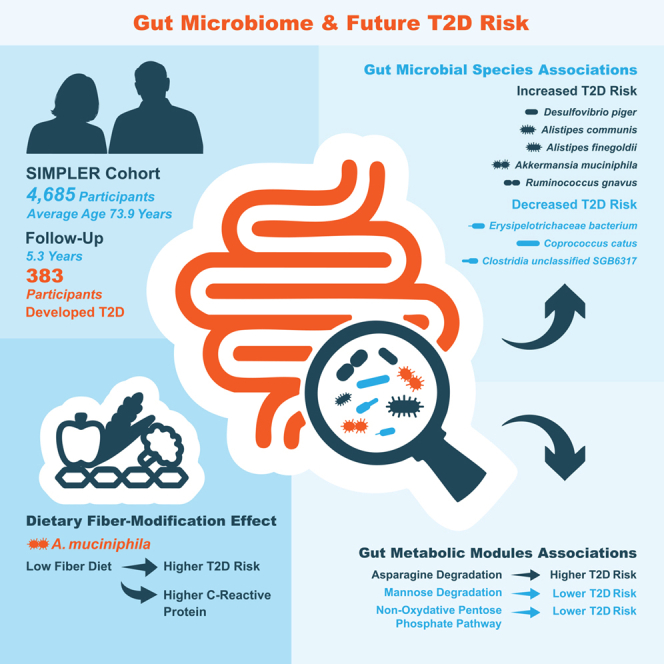

## Teaser

Toubon et al. show that the gut microbiome measured years before diagnosis predicts future type 2 diabetes risk. Using a large prospective Swedish cohort of 4,685 adults, they identify specific microbial species and metabolic pathways associated with diabetes incidence, advancing understanding of the microbiome’s role in diabetes development and prevention.

## Introduction

Type 2 diabetes (T2D) is a major global public health burden, and its prevalence is projected to increase by 61.2% until 2050, affecting more than 1.27 billion people worldwide.^1^ There is a growing recognition of the role of the gut microbiome in the pathophysiology of T2D.^2^^,^^3^^,^^4^^,^^5^^,^^6^ Currently, most of the evidence linking the gut microbiome to T2D stems from cross-sectional studies, where reduced gut microbial diversity and a lower abundance of butyrate-producing bacteria in individuals with T2D are among the most recurrent findings.^5^^,^^7^ However, due to their cross-sectional nature, these studies are limited in their ability to infer causality. Moreover, increasing evidence suggests that some of the observed associations may be confounded by the use of antidiabetic drugs that can impact the gut microbiome composition, such as metformin.^8^^,^^9^

Findings from experimental models support the role of the gut microbiome in T2D development through several interconnected pathways, including altered glucose and lipid metabolism, reduced insulin sensitivity, increased gut permeability, and immune modulation.^3^ A recurring feature across these pathways is the emergence of chronic low-grade inflammation, which contributes to insulin resistance and disease progression. Changes in gut microbiome composition, shaped by dietary and other environmental factors, appear to influence these processes.^10^ Among dietary factors, fiber intake plays a central role in modulating the gut microbiome composition and activity. Its protective effect against T2D^11^ is thought to be partly mediated by its prebiotic action on fiber-fermenting bacteria that produce short-chain fatty acids (SCFAs), such as butyrate.^12^ In contrast, low fiber intake has been linked to reduced SCFA production and impaired intestinal mucus barrier,^13^^,^^14^ which may permit microbial translocation that triggers metabolic endotoxemia ultimately promoting systemic low-grade inflammation and increasing T2D risk.

Despite mounting evidence, prospective studies investigating the gut microbiome in relation to incident T2D remain scarce. Most of the few available studies rely on 16S rRNA gene sequencing^15^ and are limited by small sample sizes,^16^^,^^17^ with inconsistent findings across studies. To date, only one large-scale prospective study using shotgun metagenomics has been published.^18^ This study identified four microbial species (including *Ruminococcus gnavus*) associated with incident T2D in a Finnish population. Besides, while these studies have investigated gut microbial composition in relation to incident T2D, only a few have comprehensively examined gut metabolites or other functional readouts in relation to T2D in a prospective setting.^19^^,^^20^^,^^21^ This limited evidence emphasizes the need for further research using large-scale datasets and more comprehensive analytical approaches.

To address these limitations, we aimed to investigate both taxonomic and functional gut microbiome features associated with T2D incidence in a T2D medication-naive population. We leveraged data from the Swedish Infrastructure for Medical Population-based Life-course and Environmental Research (SIMPLER), a well-phenotyped cohort of men and women. We used whole-genome shotgun sequencing to profile the gut microbiome in 4,685 individuals, providing an opportunity to examine microbial features in relation to incident T2D using a large-scale, prospective design.

## Results

Over a median follow-up of 5.3 years (interquartile range, 3.3–6.9 years), a total of 383 individuals (8.2%) with incident T2D were identified among 4,685 participants. This included 925 from the Swedish Mammography Cohort clinical subcohort Uppsala (SMCC-U) (mean [SD] age, 78.8 [6.6] years; 34 [3.7%] with incident T2D), 1,370 from the Swedish Mammography Cohort clinical subcohort Västmanland (SMCC-V) (mean [SD] age, 73.7 [3.2] years; 96 [6.8%] with incident T2D), and 2,390 from the Cohort of Swedish Men clinical subcohort Västmanland (COSMC-V) (mean [SD] age, 72.1 [4.9] years; 256 [10.7%] with incident T2D). The characteristics of the study participants are reported in Table 1.

**Table 1:** Baseline characteristics of the population

	Uppsala	Västmanland	
	SMCC-U (*N* = 925)	SMCC-V (*N* = 1,370)	COSMC-V (*N* = 2,390)
Women	925 (100%)	1,370 (100%)	0 (0%)
Baseline age (years)	78.8 (6.64)	73.7 (3.18)	72.1 (4.90)
Education			
<10 years	191 (20.6%)	341 (24.9%)	543 (22.7%)
10–12 years	323 (34.9%)	670 (48.9%)	1,278 (53.5%)
>12 years	411 (44.4%)	359 (26.2%)	569 (23.8%)
Height (cm)	162 (6.67)	163 (6.14)	177 (6.46)
Waist (cm)	87.7 (11.6)	89.0 (11.6)	97.4 (10.1)
Smoking status			
Non-smokers	572 (61.8%)	805 (58.8%)	1,160 (48.5%)
Current smokers	46 (5.0%)	84 (6.1%)	147 (6.2%)
Former smokers	307 (33.2%)	481 (35.1%)	1,083 (45.3%)
Total energy intake (kcal/day)	1,790 (497)	1,850 (526)	2,450 (683)
Coffee intake (cups/day)	2.51 (1.26)	2.52 (1.56)	2.80 (2.67)
Alcohol intake (g/day)	5.12 (5.57)	4.95 (5.12)	10.5 (9.29)
Walking/biking this past month			
Never	88 (9.5%)	199 (14.5%)	382 (16.0%)
<20 min	431 (46.6%)	559 (40.8%)	898 (37.6%)
20–40 min	194 (21.0%)	350 (25.5%)	558 (23.3%)
40–60 min	182 (19.7%)	202 (14.7%)	430 (18.0%)
>60 min	30 (3.2%)	60 (4.4%)	122 (5.1%)
Exercise this past month			
Almost never	352 (38.1%)	517 (37.7%)	1,184 (49.5%)
<1 h/week	87 (9.4%)	132 (9.6%)	277 (11.6%)
1 h/week	207 (22.4%)	318 (23.2%)	365 (15.3%)
2–3 h/week	233 (25.2%)	348 (25.4%)	447 (18.7%)
≥4 h/week	46 (5.0%)	55 (4.0%)	117 (4.9%)
Whole grains (g/day)	129 (81.2)	132 (84.6)	174 (105)
Yogurt (g/day)	196 (229)	202 (215)	246 (293)
Red/processed meat (g/day)	33.7 (21.4)	36.8 (23.3)	62.0 (33.3)
Sugary food/sweetened beverages (g/day)	135 (122)	128 (116)	200 (199)
Fasting plasma glucose (mmol/L)	5.55 (0.797)	5.40 (0.807)	5.67 (0.861)
Statin medication	272 (29.4%)	515 (37.6%)	949 (39.7%)

Categorical variables are presented as number of events (%), and continuous variables are presented as mean (SD).

### Gut microbiome α- and β-diversity and T2D incidence

In the full analysis set (FAS) of 4,685 participants, we observed weak and statistically non-significant inverse associations between α-diversity and incident T2D (species richness: hazard ratio [HR] = 0.999, 95% confidence interval [CI] = 0.997–1.001, *q* = 0.567; Shannon index: HR = 0.847, 95% CI = 0.659–1.09, *q* = 0.263). For β-diversity, two principal components (PCs) were statistically significantly associated with higher T2D risk (PC5: HR = 1.016, 95% CI = 1.007–1.025, *q* = 0.007 and PC6: HR = 1.013, 95% CI = 1.004–1.023, *q* = 0.028) (Table S2). However, these associations were driven to null in the lag time analysis set (LTAS) of 4,633 participants (*q* value >0.05) (Table S2).

### Gut microbiome species associations with T2D incidence

**Figure 1:**
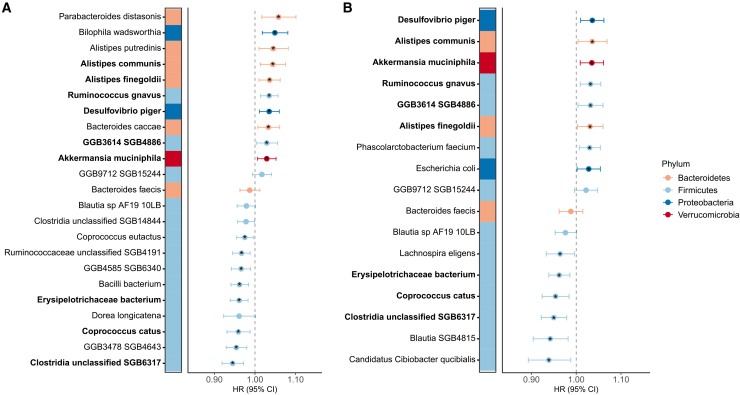
Gut microbial species associated with incident T2D Hazard ratios (HRs) of Cox regression models between (A) the 23 selected species and incident T2D after Elastic Net feature selection using the full analysis set ([FAS], *n*_total_ = 4,685 with 383 T2D incident cases) and (B) the 17 selected species and incident T2D after Elastic Net feature selection, using the lag time analysis set ([LTAS], *n*_total_ = 4,633 with 331 T2D incident cases). All models were adjusted for sex, baseline age, level of education, height, waist circumference, smoking status, walking/cycling, exercise, coffee consumption, daily intakes of total energy, alcohol, whole grains, yogurt, red/processed meat, sugary food/sweetened beverages, statin medication use, aliquoting plate, and sequencing depth. Species are colored according to their phylum, and species in bold correspond to overlapping species selected by Elastic Net and associated with incident T2D across both analysis sets. ∗*q* value < 0.05.

For species-level analyses in the FAS, 23 species were consistently selected by Elastic Net as strong predictors of future T2D development (Figures S2 and S3), suggesting that they may form a core microbiome set of particular biological relevance to T2D development. Predictive models including these 23 selected species demonstrated moderate ability to differentiate risk (mean C-index, 0.78 ± 0.02) (Figure S4). Among the 23 species selected as T2D predictors, 18 were significantly associated with incident T2D in subsequent multivariable Cox regression models (*q* value <0.05) (Figure 1A). Ten species showed a positive association and eight showed a negative association with T2D risk. Half of the high T2D risk-associated species belonged to the Bacteroidetes (Bacteroidota) phylum, while all inversely associated species belonged to the Firmicutes (Bacillota) phylum.

In the LTAS, 17 species emerged from the Elastic Net selection procedure as strong predictor of incident T2D (Figure S2). Model performance was slightly higher and showed a mean C-index of 0.81 ± 0.02, indicating good predictive performance (Figure S4)**.** Of the 17 selected species, 12 overlapped with those identified in the FAS (Figure S2), and 9 of these were associated with incident T2D in subsequent multivariable Cox regression models, supporting their robust association with incident T2D (Figure 1B).

Six species were positively associated as follows: *Desulfovibrio piger* (FAS: HR = 1.035, 95% CI = 1.011–1.06, *q* = 0.011; LTAS: HR = 1.036, 95% CI = 1.01–1.062, *q* = 0.016), *Alistipes communis* (FAS: HR = 1.044, 95% CI = 1.013–1.075, *q* = 0.011; LTAS: HR = 1.036, 95% CI = 1.004–1.069, *q* = 0.039), *Akkermansia muciniphila* (FAS: HR = 1.029, 95% CI = 1.006–1.052, *q* = 0.021; LTAS: HR = 1.035, 95% CI = 1.009–1.061, *q* = 0.016), *R. gnavus* (FAS: HR = 1.035, 95% CI = 1.014–1.057, *q* < 0.001; LTAS: HR = 1.032, 95% CI = 1.009–1.055, *q* = 0.016), GGB3614 SGB4886 (corresponding to CAG-194 sp000432915 in the Genome Taxonomy Database [GTDB],^22^ FAS: HR = 1.029, 95% CI = 1.003–1.056, *q* = 0.036; LTAS: HR = 1.032, 95% CI = 1.004–1.06, *q* = 0.039), and *Alistipes finegoldii* (FAS: HR = 1.036, 95% CI = 1.01–1.062, *q* = 0.011; LTAS: HR = 1.031, 95% CI = 1.003–1.06, *q* = 0.039). Interestingly, these overlapping species showed the strongest associations among the positively associated species in the LTAS. Three species were consistently found inversely associated with incident T2D. These species were Erysipelotrichaceae bacterium (FAS: HR = 0.961, 95% CI = 0.939–0.983, *q* = 0.004; LTAS: HR = 0.962, 95% CI = 0.939–0.986, *q* = 0.016), *Coprococcus catus* (FAS: HR = 0.959, 95% CI = 0.931–0.988, *q* = 0.011; LTAS: HR = 0.954, 95% CI = 0.924–0.984, *q* = 0.016), and Clostridia unclassified SGB6317 (FAS: HR = 0.945, 95% CI = 0.919–0.972, *q* = 0.002; LTAS: HR = 0.95, 95% CI = 0.922–0.979, *q* = 0.014).

**Figure 2:**
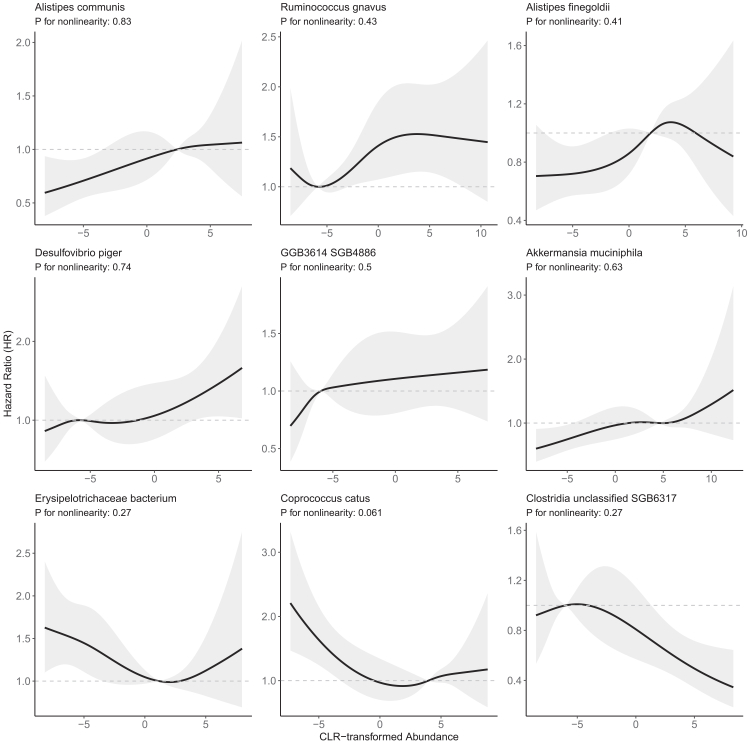
Nonlinear association between gut microbial species abundance and incident T2D Restricted cubic spline (RCS) curves from Cox proportional hazards models showing adjusted hazard ratios (HRs) for incident T2D across Centered Log-Ratio (CLR) abundance of the 9 robust species identified in both analysis sets. The solid line corresponds to the estimated HR, and the shaded band corresponds to the 95% CI. The horizontal dashed line indicates HR = 1, corresponding to the reference point set at the median abundance of each species. The *p* value for the nonlinear term (*p*-nonlinear) was estimated from a likelihood ratio test comparing the RCS term with a linear term. Models are adjusted for statin medication use, sex, baseline age, education, height, waist circumference, smoking, total energy intake, coffee, alcohol, walking/biking, exercise, whole grains, yogurt, red/processed meat, sweet foods/beverages, aliquoting plate, and sequencing depth. Knots are placed at the 5th, 35th, 65th, and 95th percentiles. The RCS is shown using data from the full analysis set (*n*_total_ = 4,685 with 383 T2D incident cases).

We further investigated species-level associations using restricted cubic splines to assess potential deviations from linearity between species abundance and T2D risk. Across the 9 robustly associated species, there was little evidence of nonlinearity (*p* for nonlinearity ≥ 0.10), except for *C. catus*, which showed borderline evidence (*p* for nonlinearity = 0.06). For *C. catus*, the decile contrasts referenced to the median showed that elevated risk was confined to the very low tail (10th percentile HR = 1.374, 95% CI = 1.059–1.784), whereas HRs from the 20th through the 90th percentiles were near unity (0.924–1.105, CIs crossing 1). This pattern suggests a low-abundance penalty with little evidence of further benefit across typical and higher abundances. Overall, within the observed distributions, associations between species abundance and T2D risk were approximately linear for most species (Figure 2). When analyses were restricted to the LTAS, similar patterns were observed (Figure S5).

**Figure 3:**
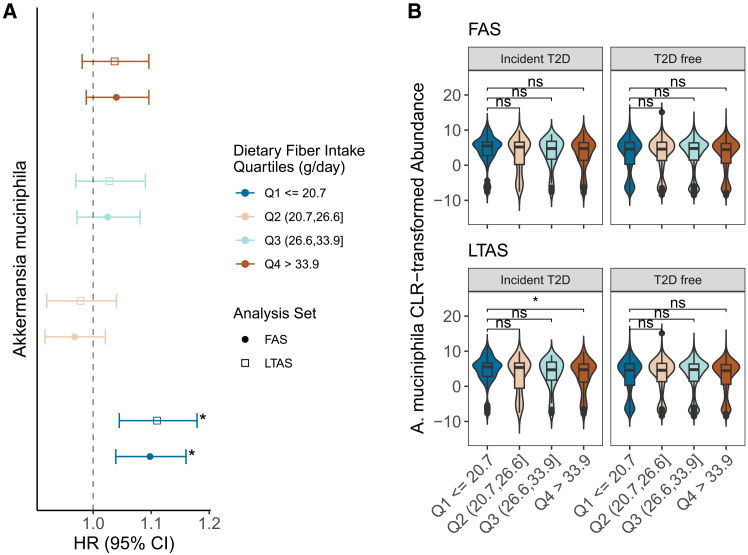
Association between *Akkermansia muciniphila*, dietary fiber intake, and incident T2D (A) HRs of Cox regression models between the *A. muciniphila* and incident T2D using the full analysis set ([FAS], *n*_total_ = 4,685 with 383 T2D incident cases) and the lag time analysis set ([LTAS], *n*_total_ = 4,633 with 331 T2D incident cases) stratified by levels of dietary fiber intake. Quartiles of dietary fiber intake are expressed as g/day. The model is adjusted for sex, baseline age, level of education, height, waist circumference, smoking status, walking/cycling, physical exercise, coffee consumption, daily intakes of total energy, alcohol, yogurt, red/processed meat, sugary food/sweetened beverages, statin medication use, aliquoting plate, and sequencing depth. ∗*p* value < 0.05. (B) Boxplot indicating the distribution of *A. muciniphila* across the four quartiles of dietary fiber intake for both the FAS and LTAS. ns, non-significant, ∗*p* < 0.05 (Student’s *t* test).

As *A. muciniphila* is known to be linked to dietary fiber intake, we further investigated the potential modifying effect of dietary fiber intake by stratifying the *A. muciniphila* model by dietary fiber intake quartiles without adjusting for whole grains. Interestingly, we observed a stronger significant effect of *A. muciniphila* on T2D risk in participants within the lowest quartile of dietary fiber intake, which corresponded to an intake of ≤20.7 g/day (FAS: Q1, HR = 1.098, 95% CI = 1.039–1.161, *p* = 0.017 vs. Q4, HR = 1.040, 95% CI = 0.988–1.096, *p* = 0.205; LTAS: Q1, HR = 1.111, 95% CI = 1.046–1.180, *p* < 0.001 vs. Q4, HR = 1.036, 95% CI = 0.980–1.095, *p* = 0.217) (Figure 3A). Among participants who developed T2D, we observed a slightly lower *A. muciniphila* abundance along higher dietary fiber intakes, but no significant differences were observed except in the LTAS, where lower abundance of *A. muciniphila* was observed at higher levels of dietary fiber intake (Q1 vs. Q4) (Figure 3B). Formal interaction tests did not indicate any statistically significant effect modification (FAS, *p*_interaction_ = 0.302; LTAS, *p*_interaction_ = 0.321), likely due to low statistical power given the low variability of *A. muciniphila* abundance across dietary fiber intake levels (Table S3). Among participants who developed T2D with C-reactive protein (CRP) data available (*n* = 381), we observed a significant interaction between *A. muciniphila* abundance and dietary fiber intake in relation to inflammation (*p* = 0.019). Opposite patterns across fiber intake levels were observed with higher *A. muciniphila* associated with increased odds of elevated CRP in a low-fiber intake context, whereas in a high-fiber intake context, it was associated with lower odds of inflammation (Figure S6), suggesting a dietary fiber intake-dependent dual role of *A. muciniphila* in relation to inflammation.

Next, we used anpan’s phylogenetic generalized linear mixed model to assess whether within-species phylogeny of *A. muciniphila* explained inter-individual heterogeneity in T2D risk. Strain-level analysis did not reveal evidence of lineage-specific associations with T2D risk. Individuals who developed T2D were distributed across the phylogenetic tree without clustering within specific clades (Figure S7), and no phylogenetic signal was detected (elpd_diff = −0.9 ± 0.3). These findings suggest that the observed positive association between *A. muciniphila* abundance and incident T2D was not driven by specific phylogenetic lineages in our cohort.

### Gut microbiome functional capability associations with incident T2D

**Figure 4:**
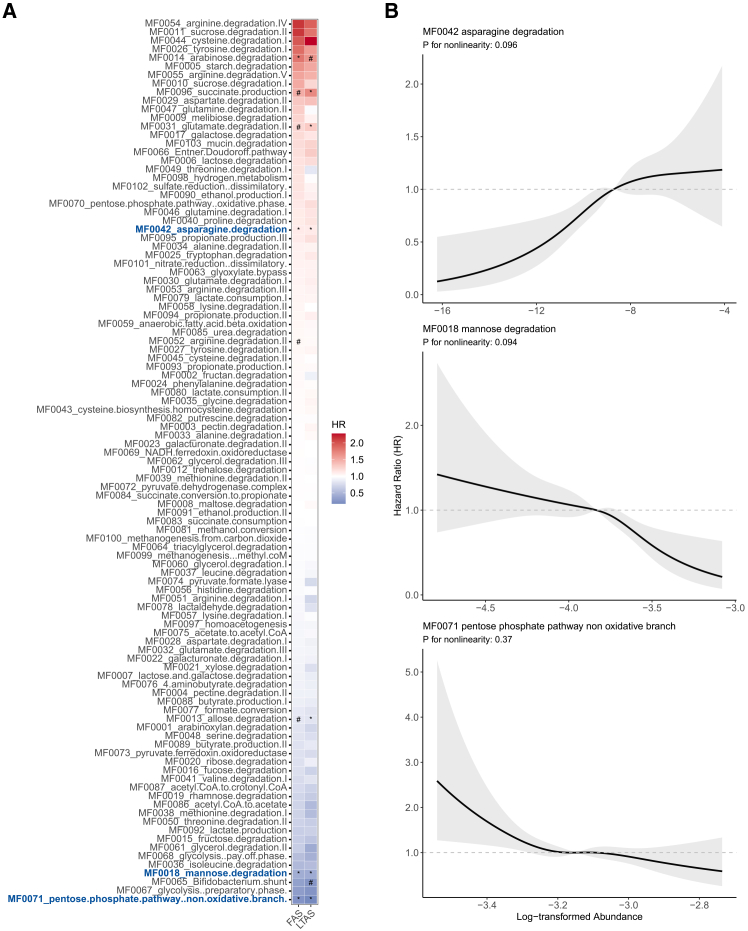
Gut metabolic modules associated with incident T2D (A) Heatmap of HRs from Cox models between GMMs and incident T2D. Results are presented for both analysis sets (FAS [full analysis set], *n*_total_ = 4,685 with 383 T2D incident cases and LTAS [lag time analysis set], *n*_total_ = 4,633 with 331 T2D incident cases). All models were adjusted for sex, age at baseline, level of education, height, waist circumference, smoking status, walking/cycling, exercise, coffee consumption, daily intakes of alcohol, whole grains, yogurt, red/processed meat, sugary food/sweetened beverages and total energy intake, statin medication use, aliquoting plate, and sequencing depth. ∗*q* value < 0.05, # 0.05 ≤ *q* value < 0.1. Robust GMMs associated with incident T2D in both analysis sets are colored in blue. (B) Restricted cubic spline (RCS) curves from Cox proportional hazards models showing adjusted hazard ratios (HRs) for incident T2D across log-transformed abundance of the three GMMs consistently associated with incident T2D. The solid line corresponds to the estimated HR, and the shaded band corresponds to the 95% CI. The horizontal dashed line indicates HR = 1, corresponding to the reference point set at the median abundance of each GMM. The *p* value for the nonlinear term (*p*-nonlinear) was estimated from a likelihood-ratio test comparing the RCS term with a linear term. Models are adjusted for the same set of covariates as the main models. Knots are placed at the 5th, 35th, 65th, and 95th percentiles. The RCS is shown using data from the FAS.

Among the 103 gut metabolic modules (GMMs) investigated, 3 GMMs were consistently associated with incident T2D across both analysis sets (Figure 4A). Asparagine degradation showed the strongest risk association (MF0042, FAS: HR = 1.122, 95% CI = 1.053–1.196, *q* = 0.014; LTAS: HR = 1.121, 95% CI = 1.046–1.201, *q* = 0.024). Two GMMs showed protective associations: mannose degradation (MF0018; FAS: HR = 0.435, 95% CI = 0.292–0.648, *q* = 0.004; LTAS: HR = 0.415, 95% CI 0.271–0.637, *q* = 0.006) and non-oxidative pentose phosphate pathway (PPP) (MF0071; FAS: HR = 0.245, 95% CI = 0.101–0.595, *q* = 0.048; LTAS: HR = 0.173, 95% CI = 0.066–0.454, *q* = 0.019). Restricted cubic splines indicated a tendency toward nonlinearity for MF0042 asparagine degradation and MF0018 mannose degradation without reaching significance (*p* for nonlinearity <0.1). For asparagine degradation, the HR increased steeply from low to mid abundance and appeared to plateau, while for mannose degradation, the HR declined with increasing abundance, with a more pronounced drop at higher levels, especially from the third quartile onward (Figure 4B). Overall, similar patterns were observed for the LTAS (Figure S8).

### Sensitivity analysis

After additional adjustment for fasting plasma glucose, several microbial features remained significantly associated with incident T2D in the FAS and the LTAS. In the FAS, *A. communis*, *R. gnavus*, Erysipelotrichaceae bacterium, Clostridia unclassified SGB6317, and the GMMs MF0018 (mannose degradation) and MF0042 (asparagine degradation) remained significantly associated with T2D risk (*q* < 0.05). In the LTAS, *A. communis*, *D*. *piger*, *A. muciniphila*, Clostridia unclassified SGB6317, and the same two metabolic modules (MF0018 and MF0042) retained significant associations with T2D risk (Table S4). The competing risk analysis,accounting for death as a competing event, yielded similar results with effect sizes comparable to those from the primary Cox regression analyses (Table S5). We replicated species-level associations in the FAS using the CHAMP taxonomic profiler. Of the nine species initially identified, eight were again selected by Elastic Net as strong predictors of T2D risk. Effect estimates were consistent in direction and magnitude across profiling methods, including for *Merdibacter merdipullorum* corresponding to Erysipelotrichaceae bacterium in MetaPhlAn (not selected by Elastic Net). Associations remained statistically significant after multiple testing correction (*q* < 0.05) for all species except *Merdicola* sp001915925 (CHAMP; corresponding to Clostridia unclassified SGB6317 in MetaPhlAn; *q* = 0.051), CAG-194 sp000432915 (CHAMP; corresponding to GGB3614 SGB4886 in MetaPhlAn; *q* = 0.056), and *Merdibacter merdipullorum* (*q* = 0.065) (Figure S9). Evidence of nonlinearity was observed for *C. catus* (*p* for nonlinearity = 0.029), consistent with the primary analysis (Figure S10).

Finally, complete case analyses indicated that our results were unaffected by confounders’ imputation as overall, similar strength, and direction of associations were observed in complete case analyses (Table S6).

## Discussion

This prospective cohort study of 4,685 individuals identified six gut microbial species and one GMM associated with a higher risk of T2D, whereas three species and two GMMs were associated with a lower risk of T2D. Gut microbiome diversity was not associated with incident T2D in our study. We comprehensively adjusted our analyses for an extensive set of potential confounding factors spanning sociodemographic, lifestyle, and dietary factors and focused on gut microbiome features that were robustly associated with incident T2D across different sensitivity analyses. Overall, our findings corroborate those of prior cross-sectional studies, while also revealing novel relationships between the gut microbiome and T2D risk. By establishing temporal associations, our results extend previous evidence, although key discrepancies with earlier work warrant careful interpretation.

*A. muciniphila*, widely recognized as beneficial for cardiometabolic health,^23^^,^^24^^,^^25^^,^^26^ showed a counterintuitive, albeit modest, positive association with T2D risk in our study. This finding contrasts with most cross-sectional studies but aligns with two prospective studies: one Finnish cohort reporting neutral associations^18^ and a European prediabetic cohort showing that an increase of *A. muciniphila* was linked to a deterioration in metabolic health.^27^ The discordance may reflect cohort-specific factors such as strain-level heterogeneity between study populations, since the above-mentioned studies were also conducted mostly in Nordic European countries, as well as the advanced age of our population and the potential effect modification of *A. muciniphila* abundance by low dietary fiber intake (≤20.7 g/day), which may contribute to context-dependent outcomes.

Although *A. muciniphila* exhibits substantial phylogenetic and functional heterogeneity across populations,^28^ with strain-specific differences in gut barrier integrity and metabolic regulation in experimental models,^29^^,^^30^ we found no evidence that strain-level variation contributed to the observed association with T2D risk in our cohort. This is consistent with prior multi-ethnic analyses showing that strain-specific associations with T2D are not uniformly observed across species; notably, some species associated with T2D at the species level do not exhibit lineage-specific effects, while strain-level signals may emerge in species without species-level associations.^31^ In the aforementioned study, most strain-level signals were heterogeneous across populations, with specific strain clusters associated with T2D enriched in particular geographic or ethnic groups, suggesting that such associations may depend on the broader microbial genetic diversity present at the population level. Our findings indicate that the relationship between *A. muciniphila* and T2D risk in our population was driven by overall species abundance rather than lineage-specific effects. Nevertheless, the potentially limited phylogenetic diversity captured within our cohort may have constrained the ability to detect strain-level signals, and this possibility cannot be excluded.

Furthermore, experimental evidence suggests that the role of *A. muciniphila* as a mucin-degrading species may also explain our findings. Murine studies have shown that dietary fiber deprivation leads to an increase in *A. muciniphila*, accompanied by greater mucous layer erosion and subsequent gut barrier dysfunction, resulting in increased susceptibility to pathogen infection.^32^^,^^33^ In our stratification analysis, the *A. muciniphila*-T2D risk association was strongest in the lowest fiber quartile (HR = 1.10 vs. 1.04 in the highest fiber quartile), supporting diet as a potential modifier of gut microbiome-T2D relationships, although formal tests for interaction did not reach statistical significance likely due to limited statistical power arising from low variability in *A. muciniphila* abundance across fiber strata in our cohort. However, among participants who developed T2D, we observed a significant interaction between *A. muciniphila* abundance and dietary fiber intake in relation to inflammation. Higher *A. muciniphila* was associated with higher odds of elevated CRP under low reported fiber intake but with lower odds under high reported fiber intake, suggesting that dietary fiber may modulate the inflammatory effects of *A. muciniphila* in line with previous evidence that dietary fiber deprivation influences its ecological niche and metabolic activity.^32^ In a low-fiber context, *A. muciniphila* compromise gut barrier integrity by thinning the mucin layer, thereby increasing intestinal permeability and promoting inflammation both locally and systemically. This is supported by human and animal studies linking microbial encroachment to chronic low-grade inflammation, insulin resistance, and dysglycemia, all hallmarks of T2D.^34^^,^^35^

Moreover, during aging, the protective function of the mucus barrier is reduced partly because of the natural decline of mucus thickness.^36^ In our elderly cohort, it is conceivable that the age-related impairment of the mucus may have contributed to the observed detrimental effect of *A. muciniphila* on T2D risk. Although evidence suggests a protective effect of *A. muciniphila* in the context of cardiometabolic diseases partially by maintaining intestinal homeostasis in the host, this protective effect may be context dependent. Under certain conditions, such as aging or low dietary fiber intake, increased *A. muciniphila* may exacerbate intestinal epithelial damage, suggesting a context-dependent, double-edged role for this bacterium.^37^^,^^38^^,^^39^^,^^40^ These context-dependent effects likely arise from interactions between microbial, dietary, and host factors that shape its ecology and function. In addition to dietary fiber, modulators such as age-related physiological changes, inflammation, and dietary components such as polyphenols can influence *A. muciniphila* abundance and activity.^28^^,^^41^ Therefore, these factors may contribute to variability across studies and highlight the importance of considering host context when interpreting its links to metabolic health.

*R. gnavus*, characterized as an inflammatory species, has been consistently linked to metabolic disorders across study designs.^18^^,^^42^^,^^43^ In our study, *R*. *gnavus* replicated its prospective association with T2D risk, aligning with Ruuskanen et al.'s Finnish cohort findings,^18^ strengthening evidence for its role in T2D pathogenesis. In the context of T2D, it has been implicated in the production of tryptamine and phenethylamine, which contribute to gut dysbiosis-induced insulin resistance.^44^ *R. gnavus* has been associated with elevated levels of imidazole propionate, a histidine-derived microbial metabolite that impairs glucose metabolism and is associated with systemic inflammation and T2D.^45^ *R. gnavus* is also linked to the production of trimethylamine N-oxide (TMAO), a gut microbiome-dependent metabolite^46^ notably associated with red meat intake^47^ that is consistently linked to increased T2D risk.^48^^,^^49^

*D. piger* has been previously associated, in cross-sectional studies, with several metabolic diseases including T2D,^50^ metabolic dysfunction-associated steatotic liver disease (MASLD),^51^ and obesity.^52^ *D. piger* is a sulfate-reducing bacterium that produces hydrogen sulfide (H_2_S) which promotes both toxic and pro-inflammatory effects for intestinal epithelial cells via activation of T helper 17 cells.^53^ Interestingly, a recent murine model study showed that *Desulfovibrio*-derived H_2_S compromises glucagon-like peptide-1 (GLP-1) production.^54^ This hormone is responsible for a variety of glucoregulatory effects, including glucose-dependent secretion of insulin and inhibition of glucagon release, which are impaired in people with T2D.^55^ Moreover, H_2_S can further facilitate the degradation of mucin by acting on the disulfide bonds,^56^ therefore, acting directly on the gut barrier integrity, which is linked to low-grade inflammation and metabolic dysregulation, as discussed above.

Two *Alistipes* species, *A. communis* (previously named *A. obesi*^57^) and *A. finegoldii*, were robustly associated with T2D risk in our study. Although recent human and experimental research suggests glucose-lowering or metabolically beneficial effects for some *Alistipes* strains,^58^ the genus shows mixed health associations,^59^ and cross-sectional studies in westernized middle-aged cohorts report inconsistent links with T2D.^60^^,^^61^ However, Mendelian randomization^62^ and animal studies^63^ support roles in insulin resistance and metabolic disruption, consistent with the pro-inflammatory properties described for *A. finegoldii*.^59^

In our study, three species were inversely associated with incident T2D: an unclassified Erysipelotrichaceae bacterium species, *C. catus*, and an unclassified Clostridia species (SGB6317).

Members of the Erysipelotrichaceae family have been linked to metabolic disorders, with decreased abundance reported in individuals with metabolic syndrome,^64^ although higher resolution analyses down to the species-level were lacking. A metagenomic study demonstrated that fecal microbiome transplants from donors with different T2D severities (mild and severe T2D) induced corresponding metabolic phenotypes in mice. Among the key species, Erysipelotrichaceae bacterium I46, which was enriched in recipients of mild T2D gut microbiome, was negatively associated with glucose tolerance parameters, suggesting a potential protective role against metabolic disturbances in the context of T2D.^65^ While species-level associations remain rare within this phylogenetic group, our findings raise the possibility that other related, yet uncharacterized, members may also contribute to metabolic health.

*C. catus* has been previously negatively associated with insulin resistance^66^ in line with its role as a butyrate-producing bacterium, whose reduced abundance is a well-established feature of T2D.^5^^,^^6^^,^^10^ SCFAs, including butyrate, improve glucose homeostasis by stimulating GLP-1 and PYY secretion, which promote satiety and improve insulin sensitivity,^5^ and by supporting gut barrier integrity, as butyrate serves as the primary energy source for colonocytes.^67^ Interestingly, a previously mentioned study^27^ showed that loss of hydrogenase-encoding bacterial genes in *C. catus* was linked to lower plasma insulin levels.^27^ Bacterial hydrogenases may play a critical role in butyrate production,^68^ which may potentially enhance host insulin sensitivity. Additionally, *C. catus* may contribute to the production of 3,4-dihydroxyphenylacetic acid (DOPAC),^69^ a phenolic compound shown to alleviate hyperglycemia and insulin resistance in T2D mouse models.^70^ For this species, we observed a potential nonlinear relationship suggesting that this species abundance range may mediate metabolic outcomes. Two additional species associated with T2D risk, one positively (GGB3614_SGB4886, Lachnospiraceae family) and one negatively (Clostridia species SGB6317, Clostridia class), have not been previously implicated in metabolic disorders. These taxa belong to species-level genome bins (SGBs), clusters of metagenome-assembled genomes grouped based on sequence similarity, often lacking formal taxonomic annotation.^71^ While MetaPhlAn4 assigns all species to SGBs, some remain labeled by their SGB identifier due to a lack of known reference genomes or taxonomic names. Their associations with T2D risk suggest that they may play previously unrecognized roles in disease pathogenesis, highlighting both the strength of shotgun metagenomics in uncovering such associations and the ongoing challenge of interpreting unannotated microbial taxa.

The asparagine degradation GMM (asparagine to aspartate) showed the strongest microbial association with T2D risk. This aligns with previous cross-sectional^72^ and prospective studies^73^^,^^74^ reporting negative associations between circulating asparagine, insulin resistance, and T2D risk, respectively, suggesting that microbial asparagine degradation may contribute to hyperglycemia and subsequent T2D. However, evidence remains conflicting, as one study linked a higher asparagine-to-aspartate ratio to increased T2D risk^75^ and its role in diabetes pathogenesis remains uncertain and warrants further investigation.

We also identified inverse associations of two functional modules with T2D risk: first, GMM MF0018, which is involved in mannose degradation (mannose-6-phosphate to fructose-6-phosphate). Mannose is considered an insulin-regulated metabolite, reflecting systemic insulin sensitivity,^76^ and elevated plasma mannose levels have been consistently linked to higher T2D and cardiovascular disease risk in prospective cohorts, independent of glycemic status.^76^^,^^77^^,^^78^ Previous studies have shown enrichment of mannose degradation genes in the gut microbiome following metformin treatment, supporting a potential protective role of microbial mannose degradation in T2D management.^79^ Our findings strengthen the hypothesis that an increased microbial mannose degradation capacity may contribute to lower circulating mannose levels, improved insulin sensitivity, and reduced risk of T2D.

Second is GMM MF0071, which represents the non-oxidative branch of PPP. The inverse association of GMM MF0071 with T2D risk may be explained by its link to SCFA production. The non-oxidative PPP contributes to bacterial carbohydrate fermentation by generating intermediates, such as pyruvate, that fuel SCFA synthesis.^80^^,^^81^ SCFAs, particularly butyrate, are known to enhance glucose homeostasis, improve insulin sensitivity, and reduce inflammation through modulation of cytokines like TNF-α and IL-6.^82^ Although individual modules involved in butyrate production showed negative but non-significant associations, the increased microbial PPP capacity observed may either better capture total SCFA synthesis, or relate to other potentially unknown mechanisms, which could support metabolic health and lower T2D risk.

Our study has several strengths, including the leverage of shotgun metagenomics that provides high taxonomic resolution in one of the largest prospective population-based cohorts to date, employing a highly standardized protocol from fecal sample collection to DNA sequencing. Incident T2D was comprehensively defined using inpatient and outpatient diagnoses alongside prescription data, offering a more objective and accurate measure than self-reports. We sought to reduce any potential reverse causation by excluding participants who developed T2D within the first year. This 1-year window removed those most likely to have marked metabolic disturbances at baseline while preserving statistical power. Although this strategy may not eliminate all potential reverse causation, it helps identify the most robust and biologically meaningful microbial associations. After further adjustment for fasting glucose, some associations weakened, suggesting early metabolic dysregulation, whereas most remained robust (*q* < 0.1), indicating long-term microbial contributors to T2D risk independent of baseline glycemia. Furthermore, our results were unaffected by antibiotic use and antidiabetic treatments, including metformin, and were not cofounded by statin medication. Additionally, for species-level analyses, we employed a robust Elastic Net resampling and cross-validation framework ensuring robust feature selection and reliable model performance evaluation. We validated the species-level associations using an independent taxonomic profiling pipeline, demonstrating robustness to bioinformatic methodology and strengthening confidence in the observed findings. Additionally, we had access to comprehensive information on potential confounders and adjusted our models for a wide range of demographic, lifestyle, and dietary factors, as well as sequencing-related technical variables.

In conclusion, our study advances gut microbiome research in T2D by identifying several gut microbial species associated with incident T2D, some of which appeared to be context and diet dependent, such as the suggested fiber-modified effect of *A. muciniphila*. By establishing temporal relationships, we extend prior cross-sectional evidence linking the gut microbiome to T2D and provide prospective validation of *R. gnavus* as a conserved microbial risk factor across populations. Moreover, we provide early evidence of putative metabolic connections between microbiome functional potential and subsequent T2D risk, years before clinical onset. If replicated, these findings could enhance risk stratification and inform personalized interventions that target diet-microbiome interactions.

### Limitations of the study

Our study also has limitations that warrant consideration. Although we adjusted for several potential cofounders throughout the modeling process, residual confounding may remain. In addition, the cohort represents an older Swedish population (mean age, 73.9 years), which may limit generalizability. Nevertheless, several associations identified in this study have also been reported in cross-sectional studies across diverse age groups and ethnic backgrounds, supporting their broader generalizability. For example, while *R. gnavus* has been linked to metabolic disorders in Norwegian adults (age range, 20–94 years),^43^ similar associations were observed in a Chinese cohort (mean age, 58 years) showing enrichment in individuals with insulin resistance and dyslipidemia.^83^ Likewise, *D. piger* has been associated with T2D in a Nigerian cohort (mean age, 57 years),^50^ and *C. catus*, inversely with insulin resistance in a Kazakh cohort (age range, 30–59 years).^66^ However, only one microbial association (*R. gnavus*) was replicated against the only large-scale reported prospective analysis to date.^18^ Discrepancies may be attributed to differences in follow-up duration and age-driven microbial shifts, as well as other differences (e.g., ethnic, cultural and dietary). Furthermore, recent evidence suggests that variations in circadian rhythm influence microbial community structure, including taxa related to glucose metabolism and T2D risk.^84^ Since stool samples in our study were collected at a single time point without standardized timing, some circadian-related variability may remain, although systematic bias is unlikely given the large, population-based design. In addition, while we propose diet-microbe interactions (e.g., fiber-*A. muciniphila*), causal mediation analyses were beyond our scope and mechanisms remain uncertain.

## Resource availability

### Lead contact

Further information and requests for resources and reagents should be directed to and will be fulfilled by the lead contact, Rikard Landberg (rikard.landberg@chalmers.se).

### Materials availability

This study did not generate new unique reagents.

### Data and code availability

The dataset supporting the conclusions of this article was provided by the national research infrastructure SIMPLER access board and cannot be shared publicly because of the sensitive nature of the data and the GDPR legislation. Data are available from the national research infrastructure SIMPLER for researchers who meet the criteria for access to confidential data. Details of obtaining data from the national research infrastructure SIMPLER can be obtained at the website www.simpler4health.se. The analysis-specific programs are publicly available under a permanent DOI in Zenodo: https://doi.org/10.5281/zenodo.19454951. Any additional information required to reanalyze the data reported in this work paper is available from the lead contact upon request.

## Acknowledgments

The authors acknowledge financial support from the project HealthFerm, which is co-funded by the 10.13039/501100000780European Union under the Horizon Europe grant agreement no. 101060247, and the Swiss State Secretariat for Education, Research and Innovation (SERI) under contract no. 22.00210. Views and opinions expressed are, however, those of the authors only and do not necessarily reflect those of the European Union or European Research Executive Agency (REA). Neither the European Union nor the REA can be held responsible for them. The authors acknowledge the SIMPLER board for enabling the current study. The computations were performed on resources provided by the National Academic Infrastructure for Super-computing in Sweden (NAISS) support for sensitive data NAISS-SENS through the 10.13039/501100015701Uppsala Multidisciplinary Center for Advanced Computational Science (UPPMAX) under project simp2023012. The 10.13039/501100004359Swedish Research Council financially supports NAISS. We acknowledge support from the Swedish Research Council (grant 2022-00924), which covered part of R.L.’s salary.

## Author contributions

G.T., C.W., and R.L. planned and designed the study. A.W. initiated the clinical subcohorts and designed feces collection. F.B. coordinated the microbiome profiling in the SIMPLER cohort. G.T. carried out statistical analyses. G.T. wrote the original draft of the manuscript with support from R.L. F.B., C.B., L.E., S.C.L., C.M.E., E.N., I.S.-K., A.W., C.W., and R.L. contributed with critical interpretation of the results for important intellectual content and reviewing and editing the manuscript. All authors read and approved the final manuscript.

## Declaration of interests

The authors declare no competing interests.

## STAR★Methods

### Key resources table

**Table:** 

REAGENT or RESOURCE	SOURCE	IDENTIFIER
**Biological samples**		

Human fecal sample	This Paper	N/A

**Deposited data**		

The analysis-specific code of this study	This paper	https://doi.org/10.5281/zenodo.19454951

**Software and algorithms**		

StaG-mwc (v0.5.1)	Boulund et al.^85^	https://stag-mwc.readthedocs.io/en/latest/index.html
StrainPhlAn (v4.0.6)	Truong et al.^86^	http://segatalab.cibio.unitn.it/tools/strainphlan/
CHAMP	Pita et al.^87^	https://cmbio.io/omics-sequencing-services/microbiome-profiling
R software (v4.3.1)	R Foundation	https://www.r-project.org
Omixer-RPM (v.0.3.2)	Darzi et al.^88^	https://github.com/raeslab/omixer-rpm?tab=readme-ov-file
vegan (v2.6–4)	CRAN	https://cran.r-project.org/web/packages/vegan/index.html
glmnet (v4.1-8)	CRAN	https://cran.r-project.org/web/packages/glmnet/index.html
anpan (v0.3.0)	Ghazi et al.^89^	https://github.com/biobakery/anpan
missForest (v1.5.0)	CRAN	https://cran.r-project.org/web/packages/missForest/index.html

### Experimental model and study participant details

The study used data from the **S**wedish **I**nfrastructure for **M**edical **P**opulation-Based **L**ife-Course and **E**nvironmental **R**esearch (SIMPLER; www.simpler4health.se) which consists of two prospective cohort studies: the Swedish Mammography Cohort (SMC) and the Cohort of Swedish Men (COSM). SMC was established in 1987–1990 and included women born between 1914 and 1948 (*n* = 90,303) and residing in the Swedish counties Uppsala or Västmanland at time of study enrollment. COSM was established in 1997, including men born between 1918 and 1952 (*n* = 100,303) residing in the counties Örebro or Västmanland at time of study enrollment.^90^ Data on diet, lifestyle, and health status from all SIMPLER participants were collected with questionnaires in 10-year intervals. In addition, data from SIMPLER participants can be linked to individual’s information from the Swedish population registries through unique personal identity numbers, providing almost complete continuously updated information on disease diagnoses and medication prescriptions for the entire study population.

A random selection of participants from each cohort were invited to take part in an extended collection of health-related information, referred to as the clinical subcohorts. For a subset of these participants, biological samples, including fecal samples, were also collected. Data collection was conducted via clinical visits in several subgroups according to the regions of residency of the cohort participants. The SMC clinical subcohort (SMCC) consists of two subgroups: SMCC Uppsala (SMCC-U, *n* = 1,539), conducted between 2012 and 2021; and SMCC Västmanland (SMCC-V, *n* = 2,630), conducted between 2010 and 2019. All COSM clinical subcohort were conducted in Västmanland from 2010 to 2019 (COSMC-V, *n* = 4,750). In total, 8,919 invited participants (60%) were included in the clinical subcohorts comprising fecal sample collection. At clinic study centers, all subcohort participants underwent extensive standardized physical examination, physiological and cognitive tests, and information on dietary habits, health status, and other health-related lifestyle information was collected with validated questionnaires.

Metagenomic analyses were conducted before sample collection was completed. At the time of analysis, of the 8,919 participants in the clinical subcohorts, 7,249 had donated fecal samples (SMCC-U, *n* = 1,464; SMCC-V, *n* = 2,245; COSMC-V, *n* = 3,540) and gut microbiome metagenomic data were available for 6,150 of them. In the present study, we excluded participants meeting one or more exclusion criteria: colorectal cancer or inflammatory bowel disease (*n* = 270), antibiotic use in the past 6 months before the clinical visit (*n* = 704, defined as a dispensed prescription, Anatomical Therapeutic Chemical code J01), and prevalent diabetes (including those identified based on T2D medication; *n* = 491). After exclusions, 4,685 participants were included in the current study.

The current study was approved by the Regional Ethical Review Board in Stockholm, Sweden under the registration number Dnr 2023-02665-02 and amendment number Dnr 2024-00303-01. All participants provided written informed consent prior to participation.

### Method details

#### Assessment of type 2 diabetes and follow-up

For all study participants, complete information on the diagnosis of T2D before or during the study period was obtained by linkage with the Swedish National Patient Register (NPR), which contains inpatient and outpatient specific diagnostic data from the Swedish healthcare system. T2D was defined according to ICD-10 code E11, ICD-9 code 250, and ICD-8 code 250. We further identified prevalent and incident cases using the Swedish Prescribed Drug Register targeting prescriptions of drugs used in diabetes (ATC A10) which includes metformin, reported to have an effect on the gut microbiome.^8^^,^^9^ All participants with a T2D identification date before the individual fecal sample collection date were defined as prevalent T2D case in this study, and all participants with a T2D identification date between fecal sample collection and study censoring date were defined as incident T2D cases. For prevalent cases only, register data were supplemented with measured fasting plasma glucose concentrations of ≥7.0 mmol/L according to WHO guidelines.

Dates of death were obtained from the Swedish Death Register. In prospective analysis with T2D incidence as outcome, for participant under risk (free of T2D at baseline), follow-up time accrued from fecal sampling date until the date of T2D diagnosis, death, or the censoring date of the study (31 December 2021), whichever came first.

#### Microbiome data

Participants received a pre-packaged fecal sample collection kit including instructions on how to collect the sample at home. The participants were asked to store the samples at −20°C in the home freezer at least 24h before the clinical visit. Once received at the clinical visit, samples were aliquoted with DNA/RNA shield (Zymo Research) and stored at −80°C until shipment on dry ice to the Center for Translational Microbiome Research (CTMR) at the Karolinska Institutet (Stockholm, Sweden) for DNA extraction.

DNA extraction was performed using MagPure Stool DNA LQ kit from Magen Biotechnology Co, Ltd. Briefly, 800 μL of the fecal samples were transferred to cryo tubes. A negative control (800 μL DNA/RNA) shield and a positive control (75 μL Zymo mock) were included. The samples were centrifuged at 14 000 g for 25 min and the supernatant was removed. To each tube, 600 μL ATP/PVP, 600 μl PCI and MagPure bead were added. The samples were bead beaten in a FastPrep 96 at 1600 rpm for 1 min. The samples were incubated at 65°C for 20 min and thereafter centrifuged for 3 min at 14 000 x g. Then, 340 μL of the upper phase of each sample was transferred to a deep-well plate and placed in an SP960 (MGI, Shenzhen). The following reagent plates were prepared and placed in an SP960, RNaseA 10 μL/well (15 mg/mL), Reagent mix 640 μL/well (MagPure Particles *N* 30 μl, Proteinase K (20 mg/mL) 20 μl and Buffer MLE 590 μl), GW1 (650 μL/well), 75% Ethanol (1.1 mL/well) and EB buffer (100 μl/well). The DNA was stored at −20°C. The DNA was then shipped on dry ice to MGI facilities in Riga, Latvia (Latvia MGI Tech).

The genomic DNA was subjected to library preparation using MGI’s FS library prep set as per the manufacturer’s protocol with 50 ng of input DNA. Quality assessment of the prepared libraries was performed using the TapeStation D1000 kit from Agilent, USA, while the quantity was determined by QuantIT HighSensitivity dsDNA Assay on a Tecan Spark (Tecan, Switzerland). The equimolarly pooled libraries were circularized using MGI Easy Circularization kit (MGI Tech) and subjected to DNBseq 2 × 100bp paired-end sequencing on the DNBSEQ G400 or T7 sequencing instrument (MGI) following the manufacturer’s instructions. The sequencing generated a median read count of 51.8 million (IQR = 34.8–61.2 million) read-pairs.

Sequencing data from the samples were processed with StaG-mwc, a reproducible Snakemake workflow for metagenomic analysis, version 0.5.1.^85^ Tools used in the workflow included fastp v0.23.0 for quality control and filtering, Kraken2 v2.1.2 executed with “--confidence 0.1” and “--quick” for host removal using human genome version GRCh38, and MetaPhlAn v4.0.3 was used for taxonomic profiling, yielding a species-level relative abundance table comprising 2,470 bacterial species. All tools were run with their default settings.

Strain-level profiling of *Akkermansia muciniphila* (SGB9226), was performed using StrainPhlAn v4.0.6 and the mpa_vJan21_CHOCOPhlAnSGB_202103 database. Among 171 clade-specific marker genes, 169 passed filtering and were retained. Marker sequences were trimmed by 50 bp at both ends, and samples and markers were filtered by requiring ≥80% marker presence across samples and ≥80% marker coverage per sample based on StrainPhlAn default parameters. After filtering, 3,156 of the 6,150 initial metagenomic samples were retained for phylogenetic analysis, of which 2,425 were included in the present study. StrainPhlAn uses the marker genes defined in the database to identify polymorphisms per sample, and alignments were subsequently used to infer a maximum-likelihood phylogenetic tree using the PhyloPhlAn pipeline in fast mode with RAxML.

The SIMPLER raw sequencing data were also processed at Cmbio using the CHAMP profiler^87^ as previously described elsewhere,^91^ with taxonomic annotation based on the Genome Taxonomy Database (GTDB) release 214. Species with matching taxonomic labels were directly aligned, while SGBs without species names were mapped to GTDB taxonomy using the bioBakery SGB2GTDB translation file, enabling consistent species-level annotation across methods. The functional potential of the gut microbiome was characterized using gut metabolic modules (GMMs). Catalog genes were mapped to the curated GMMs v1.07 database^92^ using EggNOG-mapper-software v2.0.1. To calculate the modules abundances, Omixer-RPM v0.3.2 was used with a minimum module coverage of 66.6%.

#### Covariates

Covariate information was obtained from either the questionnaire at the clinical examination visit, clinical measurements or register data. Assessment of anthropometric measurements was performed by the nurse during the clinic visit and included height (cm) and waist circumference (cm). Sociodemographic and lifestyle information retrieved from the questionnaire at the clinical visit included age at baseline (years), level of education, smoking status, walking/cycling and exercise during the past month. Dietary assessment was also retrieved from the questionnaire at the clinical visit and included consumption of wholegrains (g/day), yogurt (g/day), red/processed meat (g/day), sugary food/sweetened beverages (g/day), coffee (cups/day), alcohol (g/day), and total daily energy intake (kcal/day). Total energy intake was calculated by multiplying the age-specific portions by consumption frequency of each food item with the nutrient content obtained from the Swedish Food Database.^93^ Daily intake of alcohol, wholegrains, yogurt, red/processed meat, and sugary food and sweetened beverages were estimated from a validated food frequency questionnaire included in the clinical examination visit questionnaire. Wholegrains, yogurt, red/processed meat, and sugary food and sweetened beverages intakes were calculated using different items in the FFQ (Table S1). Information on blood lipid modifying drug prescriptions (statin medication) was retrieved from the Swedish Prescribed Drug Register using Anatomical Therapeutic Chemical (ATC) code C10.

### Quantification and statistical analysis

The microbiome α-diversity was estimated using the Shannon diversity index, and richness (number of different species). β-Diversity was estimated using Aitchison distance (vegan v2.6–4). Principal Component Analysis (PCA) was performed and the PCs explaining more than 1% of total variation were retained for downstream analyses leading to the inclusion of the first 6 PCs explaining together 18.9% of the total variation in the gut microbiome (Figure S1). Diversity metrics were calculated on the whole dataset without prior filtering. For downstream analyses at species level, we filtered out species that were not detected at a minimum relative abundance of 0.1% in at least 10% of samples yielding 193 species that met the criteria. Species relative abundances were then Centered Log-Ratio (CLR) transformed. For the functional potential of the gut microbiome, GMM abundances were log transformed prior to analysis.

The associations between gut microbiome features and the risk of developing T2D were estimated as hazard ratios (HRs) with 95% confidence intervals (CIs) using multivariable-adjusted Cox proportional hazards regression models with age as the underlying timescale. We constructed models for alpha diversity (species richness and Shannon index), beta diversity (6 first PC axes), species CLR transformed abundances and GMMs log transformed abundances. Models were all adjusted for the covariables mentioned above and two metagenome sequencing-related technical variables, i.e., aliquoting plate and sequencing depth.

To balance the control of false positive findings while maintaining sensitivity for detecting biologically relevant species associations, we employed a two-stage approach: first using penalized Cox regression approach with Elastic net regularization and cross-validation to identify a subset of species with collective predictive value for T2D risk, followed by individual hypothesis testing on this reduced set with appropriate multiple testing correction. To ensure robustness, we performed 10 iterations of stratified resampling (70% training, 30% testing), preserving the initial case:control ratio in each split. For each resample, penalized Cox regression models were fitted using the Elastic Net framework with a 10-fold cross-validation to optimize the combination of alpha (mixing parameter) and lambda (regularization parameter) (glmnet v4.1-8). Model performance was evaluated on the test set using the Harrell’s C-index, and species selected across resamples were summarized with their selection frequency. Species selected in at least 6 out of the 10 resamplings were considered as strong predictors of T2D risk and used for downstream analyses.

The study outcome T2D was primarily identified through register data, which raises the possibility that some participants may have had subclinical dysglycemia or undiagnosed T2D at the time of fecal sampling which may have influenced their gut microbiome.^60^ Hence, we replicated all analyses including for the species-level association, the feature selection and the Cox regression models as lag time analysis, excluding 52 participants who developed T2D within the first year of follow-up. Therefore, our analyses were performed in two analysis sets in the current study: the “*Full Analysis Set*” (FAS, n_total_ = 4,685 with 383 T2D incident cases) and the “*Lag Time Analysis Set”* (LTAS, n_total_ = 4,633 with 331 T2D incident cases) and only gut microbiome features consistently associated with incident T2D across the two analysis sets were considered as robust gut microbiome features associated with T2D risk. For these robust features, we further modeled the associations with restricted cubic splines curves (RCS) to examine whether there was a non-linear relationship between the feature and T2D risk. For all RCS terms, we pre-specified four knots placed at the 5th, 35th, 65th, and 95th percentiles of the feature’s distribution. We evaluated departures from linearity using a likelihood-ratio test (LRT) comparing the spline specification with a model containing only the linear term for the feature.

To test for statistical interaction between dietary fiber intake and *A. muciniphila* in relation to T2D risk, we compared Cox regression models with the same covariate adjustment as the main models except for wholegrain intake with and without the interaction term, using LRTs. The LRT *p*-value was used to assess whether inclusion of the interaction term significantly improved model fit, indicating evidence of interaction. To investigate strain-level variation in *A. muciniphila* associated with T2D, we applied phylogenetic generalized linear mixed models (PGLMM) using anpan^89^ to identify a phylogenetic signal related to specific health outcomes as previously reported.^31^^,^^94^ In this framework, a phylogenetic term is incorporated as a sample-specific random effect whose covariance structure is derived from the phylogenetic tree, allowing closely related strains to have correlated effects. PGLMMs are fitted in a Bayesian framework using either binomial or Gaussian likelihoods depending on the outcome variable. Here, we modeled incident T2D as a binary variable (incident T2D vs. non-T2D during follow-up), and the phylogeny as the independent variable with sex, baseline age, waist circumference, dietary fiber intake, and statin medication use included as covariates. Two models were fitted: one including the within-species phylogenetic random effect and a null model excluding the phylogenetic term but retaining all other covariates. Model performance was evaluated using leave-one-out cross-validation with Pareto-smoothed importance sampling to estimate the expected log pointwise predictive density (ELPD). The difference in ELPD between the phylogenetic and null models (elpd_diff), together with its standard error (se), was used to assess whether incorporating phylogenetic structure improved prediction of the T2D outcome. A significant phylogenetic signal was defined as an absolute difference in ELPD (|elpd_diff| ≥ 4) with confidence interval of the elpd_diff not overlapping zero (elpd_diff ± 2 SE), indicating improved predictive performance with inclusion of phylogeny as previously reported.^31^^,^^94^

For sensitivity analyses, we further adjusted the Cox regression models for fasting plasma glucose to assess whether the associations between the robust features and incident T2D were influenced by baseline glycemic status. Furthermore, Cox regression models are not designed to account for the competing risk of death, and thus, they can overestimate risk of disease in elderly individuals with high mortality rate.^95^^,^^96^ Given the relatively advanced age of the study population and that a substantial part of the participants died during the follow-up (*n* = 387), we additionally performed a competing risk analysis using Fine–Gray subdistribution hazard models, treating death as a competing event and reporting subdistribution hazard ratio (SHRs). We further replicated the species-level associations using the CHAMP profiled data. Finally, the overall proportion of missing data in the cohort was 2.3%, and missing values for covariates were imputed using a random forest-based algorithm, missForest (v1.5.0). To assess the potential impact of this imputation, we conducted a complete case analysis including only participants without missing data for covariates.

All *p-*values from high dimensional analyses were adjusted for multiple comparisons using the Benjamini–Hochberg procedure with a target rate of 0.05 for q*-*values. All statistical analyses were conducted using the R software version 4.3.1 (R Foundation).

## Supplemental information

Document S1. Figures S1–S10 and Tables S1–S6

Document S2. Article plus supplemental information
