# Supplementary material for: Gut microbiome composition and functional potential associate with incident type 2 diabetes in 4,685 adults from a Swedish prospective cohort
Source: Cell Rep Med. 2026 May 27;7(6):102835. doi: 10.1016/j.xcrm.2026.102835 (PMC13293967; doi:10.1016/j.xcrm.2026.102835)
Supplement: Document S1. Figures S1–S10 and Tables S1–S6 [file mmc1.pdf]

Cell Reports Medicine, Volume *7*

Supplemental information

Gut microbiome composition and functional potential associate with incident type 2 diabetes in 4,685 adults from a Swedish prospective cohort

Gaël Toubon, Fredrik Boulund, Cecilia Martinez Escobedo, Carl Brunius, Lars Engstrand, Susanna C. Larsson, Elise Nordin, Ina Schuppe-Koistinen, Alicja Wolk, Clemens Wittenbecher, and Rikard Landberg

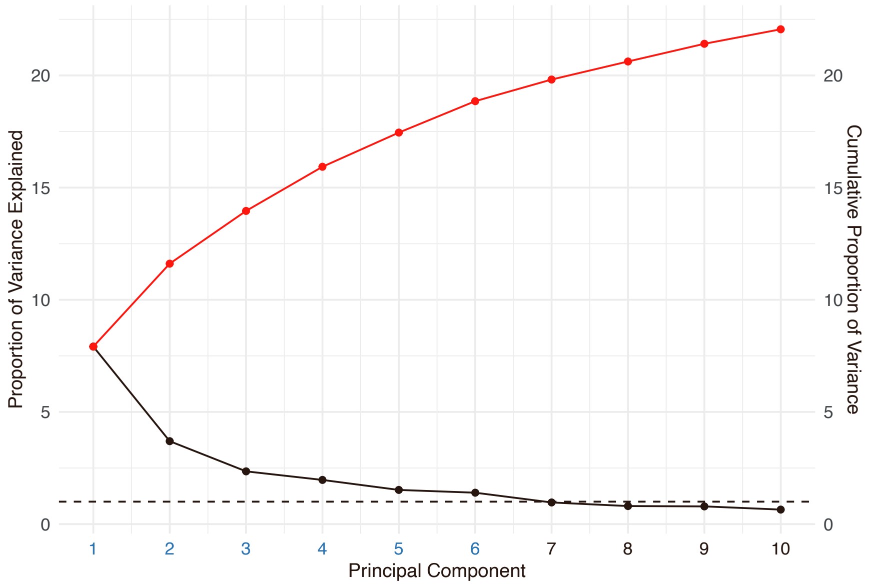

Figure S1. Percentage of variance explained across the 10 first Principal Components (PCs) after PCA analysis based on Aitchison distance using the Full Analysis set. Related to STAR Methods. PCs marked in blue explained each more than 1% in total variance. The black line corresponds to the proportion of Variance explained by each PCs while the red line corresponds to the cumulative variance explained across the 10 first PCs. The dashed line corresponds to the 1% variance explained threshold.

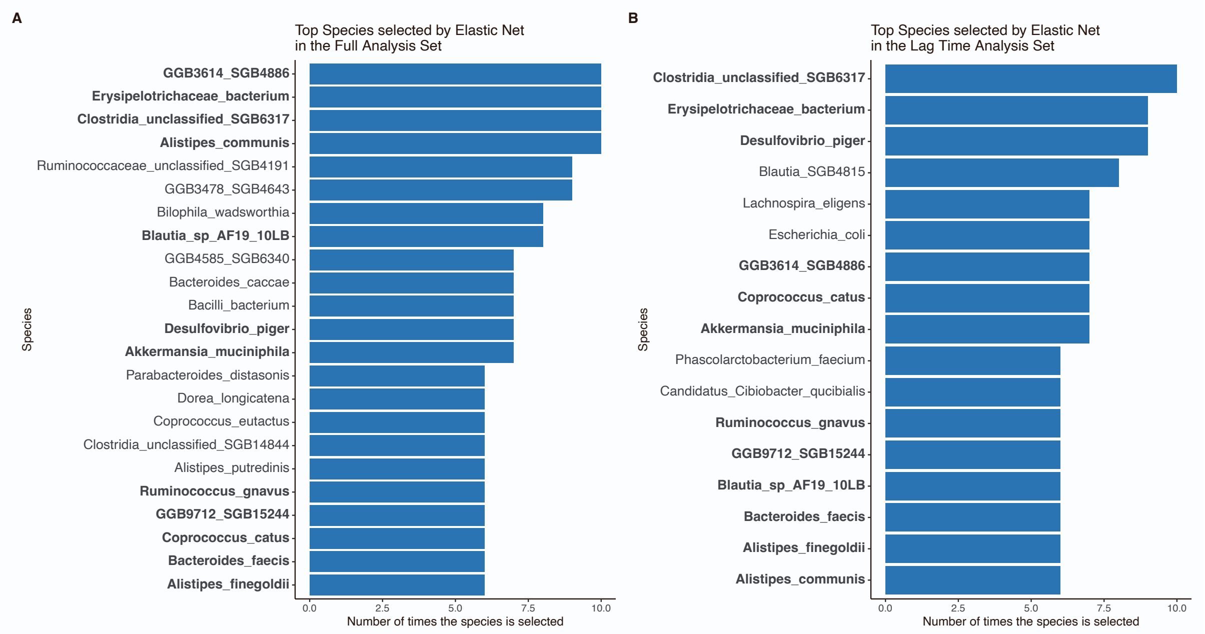

Figure S2. Species selected at least 6 times across 10 random resampling 10-fold cross validation Elastic Net. Related to Figure 1. Overlapping selected species between **(A)** the Full Analysis Set (n_{total}=4,685 with 383 T2D incident cases) and **(B)** the Lag Time Analysis Set (n_{total}=4,633 with 331 T2D incident cases) are marked in bold.

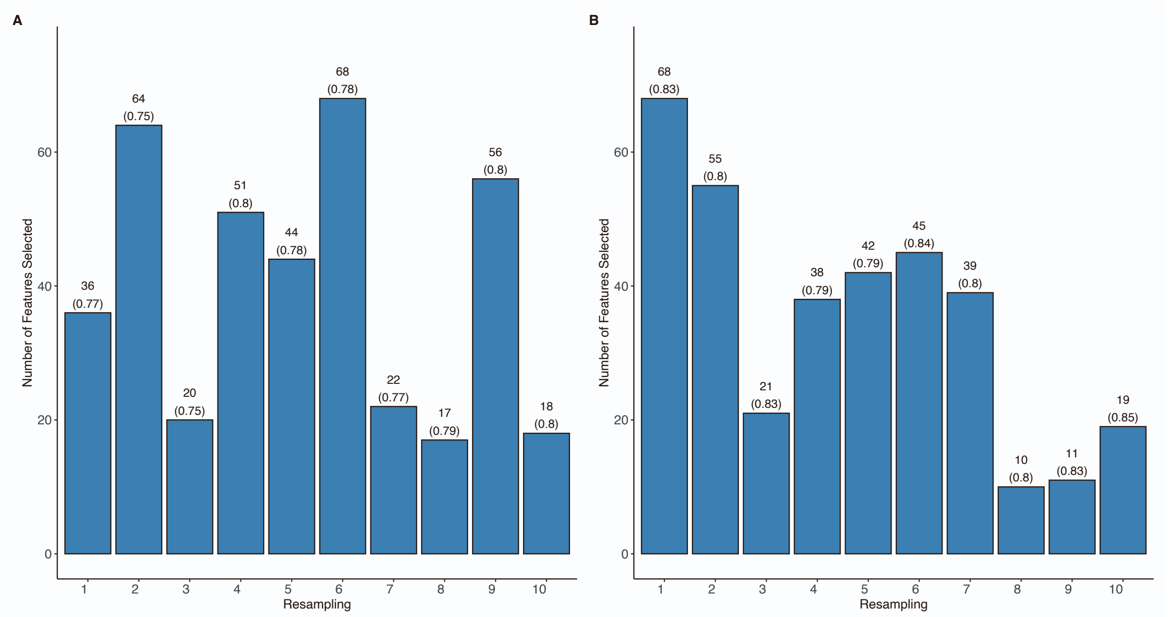

Figure S3. Number of species selected across each resample in (A) the Full Analysis Set and (B) the Lag Time Analysis Set. Related to Figure 1. Number on top of the bars corresponds to the number of species selected while number in parentheses corresponds to the C-index evaluated on the test set for each resample.

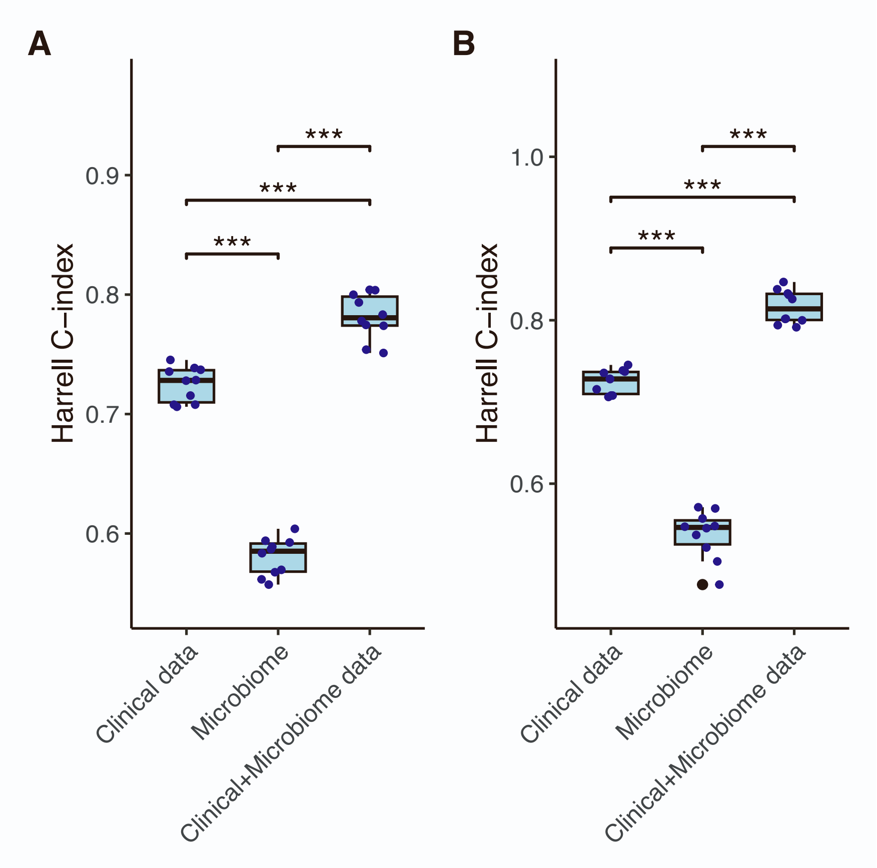

Figure S4. Feature selection model performance based on Harell c-index across the 10 resamples from penalized Cox regression with Elastic Net regularization models using either clinical data (covariates used for model adjustment, see covariates paragraph in the methods section) alone, microbiome data alone (species CLR transformed abundances) or a combination of clinical and microbiome data in (A) the Full Analysis Set and (B) the Lag Time Analysis Set. Related to Figure 1. For each resample, model performance was evaluated on the test set. *** $p < 0.001$

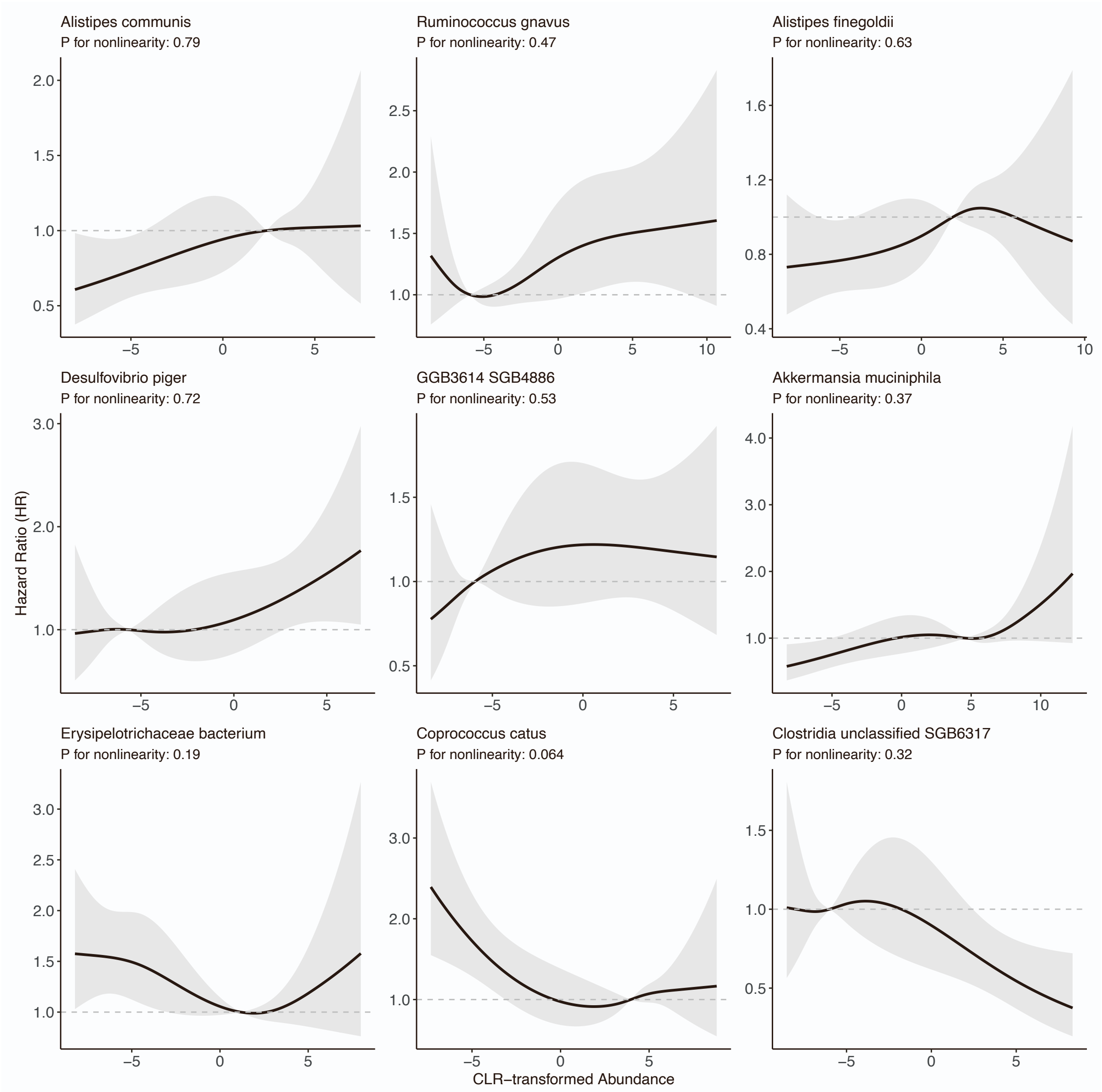

Figure S5. Restricted cubic spline (RCS) curves from Cox proportional hazards models from the LTAS showing adjusted hazard ratios (HR) for incident T2D across centered log-ratio (CLR) abundance of the 9 robust species identified in both analysis sets. Related to Figure 2. The solid line corresponds to the estimated HR and the shaded band the 95% CI. The horizontal dashed line indicates HR = 1, corresponding to the reference point set at the median abundance of each species. The p-value for the nonlinear term (P-nonlinear) was estimated from a likelihood-ratio test comparing the RCS term with a linear term. Models are adjusted for statin medication use, sex, baseline age, education, height, waist circumference, smoking, total energy intake, coffee, alcohol, walking/biking, exercise, whole grains, yogurt, red/processed meat, and sweet foods/beverages, aliquoting plate, and sequencing depth. Knots are placed at the 5th, 35th, 65th, and 95th percentiles. The RCS are shown using data from the Lag Time Analysis Set (n_{total}=4,633 with 331 T2D incident cases).

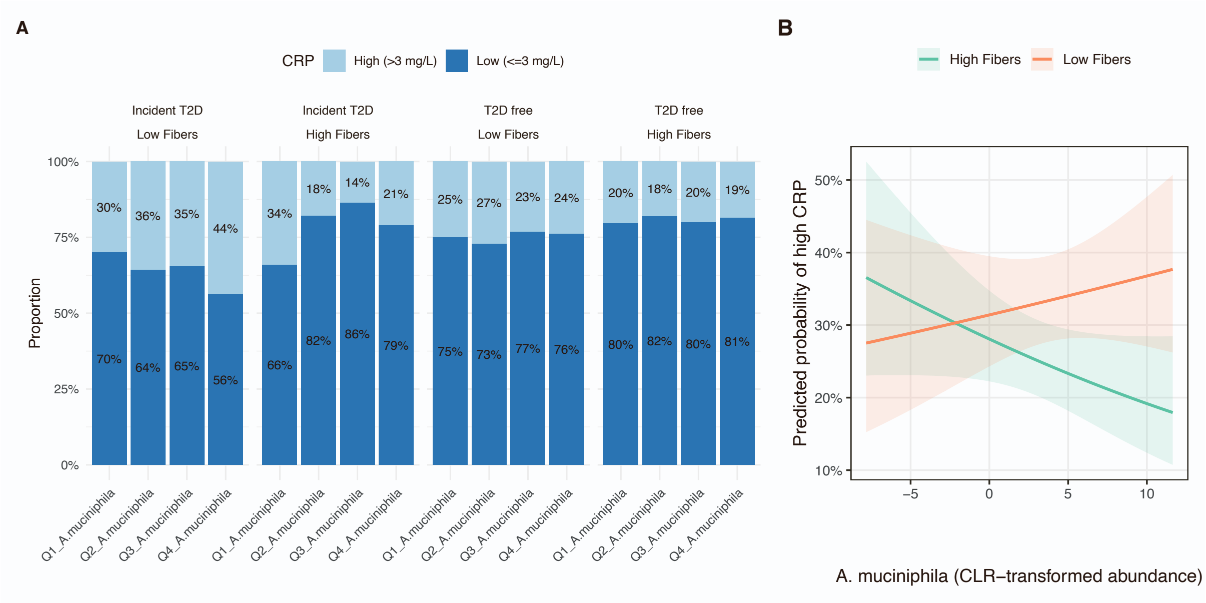

Figure S6. *Akkermansia muciniphila* and inflammation in different dietary fiber intake contexts. Related to Figure 3. (A) The barplots show the proportion of high and low CRP using 3 mg/L as threshold in high or low dietary fiber contexts (median distribution threshold) across quartiles *of A. muciniphila* abundance stratified by T2D status (n=4,649). **(B)** Predicted probability of high CRP by *A. muciniphila* abundance in high and low dietary fiber contexts in participants who developed T2D during the follow-up (n=381). A logistic regression model was first fitted with high CRP as the binary outcome and included *A. muciniphila*, dietary fiber intake, and their interaction term. Participants were divided into two groups based on the median dietary fiber intake (low vs. high fiber). Predicted probabilities of high CRP were then estimated across the observed range of *A. muciniphila* abundance, holding dietary fiber constant at the median value within each group. The resulting curves, with 95% confidence intervals, were plotted to illustrate how the association between *A. muciniphila* and inflammation differed between low- and high-fiber contexts.

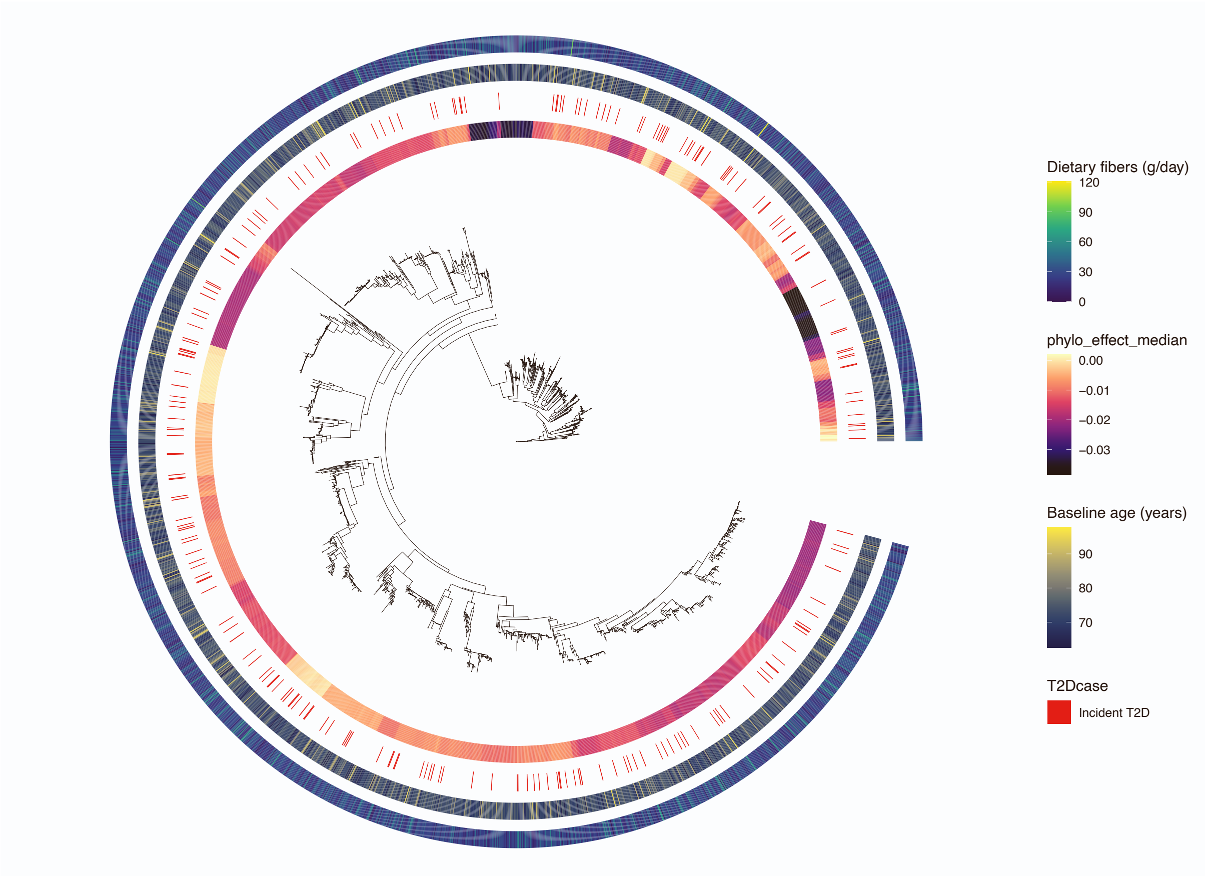

Figure S7: Phylogenetic tree of *Akkermansia muciniphila* in relation to T2D outcome. Related to Figures 1 and 3. Gut metagenomic samples from 2,425 individuals, of which, 213 developed T2D during the follow-up (red annotation) showing no strong phylogenetic signal for the T2D outcome. The phylogenetic effect median (phylo_effect_median) indicating the strength of the phylogenetic signal on T2D prediction per sample adjusted for sex, baseline age, waist circumference, dietary fibers, and statin medication. Only, baseline age, and dietary fibers are annotated in the outer rings.

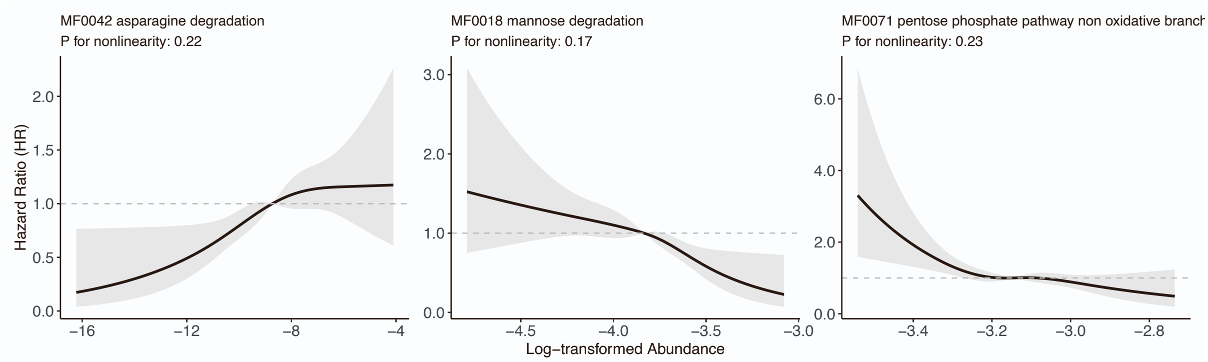

Figure S8. Restricted cubic spline (RCS) curves from Cox proportional hazards models from the LTAS showing adjusted hazard ratios (HR) for incident T2D across log-transformed abundance of the three GMMs consistently associated with incident T2D. Related to Figure 4. The solid line corresponds to the estimated HR and the shaded band the 95% CI. The horizontal dashed line indicates HR = 1, corresponding to the reference point set at the median abundance of each GMM. The p-value for the nonlinear term (P-nonlinear) was estimated from a likelihood-ratio test comparing the RCS term with a linear term. Models are adjusted for statin medication use, sex, baseline age, education, height, waist circumference, smoking, total energy intake, coffee, alcohol, walking/biking, exercise, whole grains, yogurt, red/processed meat, and sweet foods/beverages, aliquoting plate, and sequencing depth. Knots are placed at the 5th, 35th, 65th, and 95th percentiles.

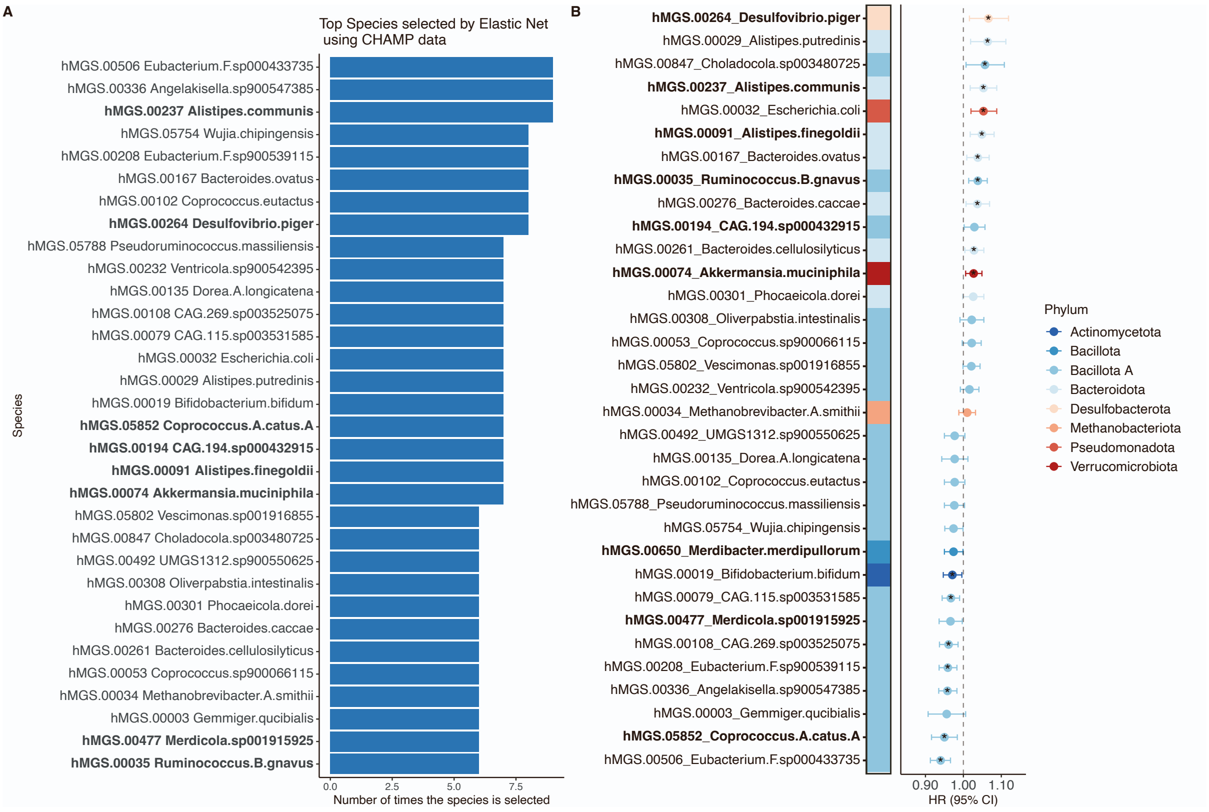

Figure S9. Replication of the species-level association using CHAMP taxonomic profiler. Related to Figure 1. (A) Species selected at least 6 times across 10 random resampling 10-fold cross validation Elastic Net. **(B)** HRs of Cox regression models between the 32 selected species by Elastic Net + *Merdibacter merdipullorum* and incident T2D. The analysis was done using the Full Analysis Set (n_{total}=4,685 with 383 T2D incident cases) and all models were adjusted for sex, baseline age, level of education, height and waist circumference, smoking status, walking/cycling, exercise, coffee consumption, daily intakes of total energy, alcohol, wholegrains, yogurt, red/processed meat, sugary food/sweetened beverages, statin medication use, aliquoting plate, and sequencing depth. Species are colored according to their phylum, and species in bold correspond to the CHAMP-labeled strong species predictors identified in the primary analysis using MetaPhlAn taxonomic profiling data. * q-value < 0.05.

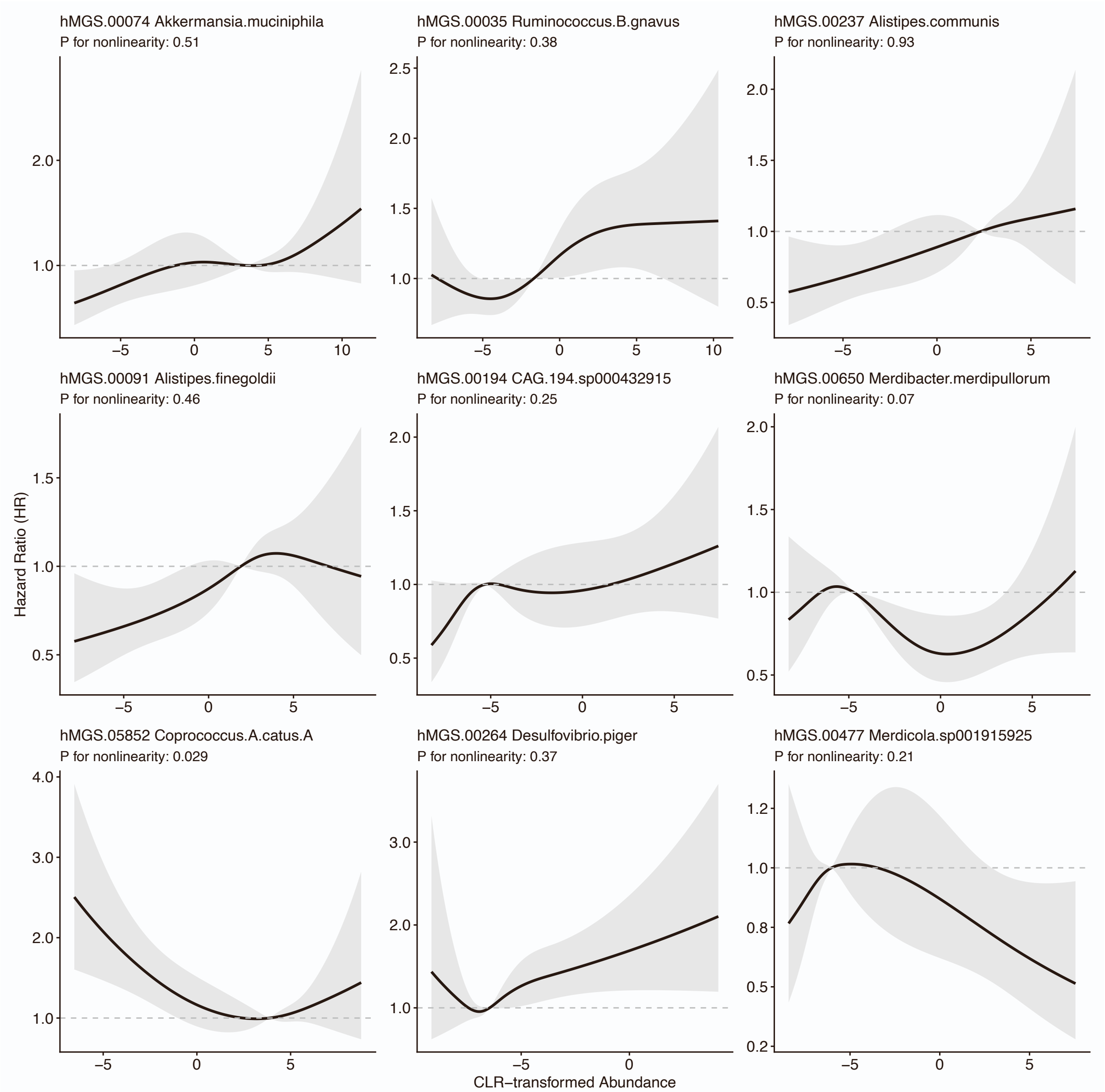

Figure S10. Restricted cubic spline (RCS) curves from Cox proportional hazards models showing adjusted hazard ratios (HR) for incident T2D across centered log-ratio (CLR) abundance of the 9 robust species identified in primary analysis using CHAMP taxonomic profiling data. Related to Figure 2. The solid line corresponds to the estimated HR and the shaded band the 95% CI. The horizontal dashed line indicates HR = 1, corresponding to the reference point set at the median abundance of each species. The p-value for the nonlinear term (P-nonlinear) was estimated from a likelihood-ratio test comparing the RCS term with a linear term. Models are adjusted for statin medication use, sex, baseline age, education, height, waist circumference, smoking, total energy intake, coffee, alcohol, walking/biking, exercise, whole grains, yogurt, red/processed meat, and sweet foods/beverages, aliquoting plate, and sequencing depth. Knots are placed at the 5th, 35th, 65th, and 95th percentiles. The RCS are shown using data from the Full Analysis Set (n_{total}=4,685 with 383 T2D incident cases).

Table S1. FFQ items used for the construction of dietary variables. Related to STAR Methods.

FFQ Sugary food/Swetened beverages variables	FFQ Red/Processed variables	FFQ Wholegrains variables	FFQ Yogurt variables
**Sugary food**	**Red meat**	Wholegrain pasta/spaghetti/macaroni	Sour milk/Yogurt (≥ 3% fat)
Sugar	Pork (steak/casserole)	Wholegrain rice (råris)	Low-fat Sour milk/Yogurt (0.5% fat)
Honey	Beef/veal (steak/casserole)	Whole grain/meal bread	Fruit flavoured Yogurt/Sour milk
Buns/cookies	Minced meat dishes (Meatballs, hamburger, minced meat sauce)	Crisp bread/Hard bread	
Biscuits/wafers	**Processed meat**	Oatmeal porridge	
Cakes/pastries	Falukorv (sausage)	Muesli	
Fruit soup/kissel	Liver paté		
Lingonberry jam	Liver paté (low-fat)		
Other jam	Cold cut meats (e.g. ham/turkey)		
Chocolate	Cold cut sausages (e.g. salami)		
Candy (not chocolate)			
Ice cream			
**Swetened beverages**			
Orange/grapefruit juice			
Coca Cola/Pepsi			
Other Soda/Squash (carbonated/non-carbonated)			

Each variable was derived as g/day.

Table S2. Alpha and Beta diversity association with incident T2D in the Full Analysis Set and the Lag Time Analysis Set. Related to Results.

	**Full Analysis Set**			**Lag Time Analysis Set**		
**Diversity metrics**	HR 95% IC	p-value	q-value	HR 95% IC	p-value	q-value
PC1	1.004 (95% CI, 1-1.008)	0.072	0.193	1.004 (95% CI, 1-1.009)	0.065	0.129
PC2	1.001 (95% CI, 0.995-1.007)	0.771	0.771	1 (95% CI, 0.994-1.007)	0.982	0.982
PC3	0.994 (95% CI, 0.987-1.002)	0.164	0.263	0.996 (95% CI, 0.987-1.004)	0.311	0.355
PC4	0.993 (95% CI, 0.985-1.001)	0.105	0.210	0.991 (95% CI, 0.982-1)	0.051	0.129
PC5	1.016 (95% CI, 1.007-1.025)	**0.001**	**0.007**	1.012 (95% CI, 1.002-1.022)	**0.018**	0.129
PC6	1.013 (95% CI, 1.004-1.023)	**0.007**	**0.028**	1.011 (95% CI, 1.001-1.022)	**0.033**	0.129
Shannon	0.847 (95% CI, 0.659-1.09)	0.197	0.263	0.813 (95% CI, 0.619-1.068)	0.137	0.218
Species richness	0.999 (95% CI, 0.997-1.001)	0.496	0.567	0.999 (95% CI, 0.997-1.001)	0.282	0.355

Cox regression models adjusted for sex, baseline age, level of education, height and waist circumference, smoking status, walking/cycling, physical exercise, coffee consumption, daily intakes of total energy, alcohol, wholegrains, yogurt, red/processed meat, sugary food/sweetened beverages, statin medication use, aliquoting plate, and sequencing depth. p-values and q-values < 0.05 are marked in bold.

Table S3. Interaction analysis of *Akkermansia muciniphila* and dietary fiber intake on in Cox proportional hazards models. Related to Figure 3.

Analysis set	exposure	interaction_var	lrt_p_value	aic_no_int	aic_with_int	int_var_type	int_var_category	int_term_1	int_p_value
FAS	*A. muciniphila*	dietary_fibers	0.538	5248.107	5249.729	continuous	NA	*A. muciniphila*:dietary_fibers	0.535
	A. muciniphila	dietary_fibers_Q	0.302	5249.310	5251.665	categorical	Q1, Q2, Q3, Q4	*A. muciniphila*:dietary_fibers _Q2	0.058
								*A. muciniphila:*dietary_fibers _Q3	0.401
								*A. muciniphila:*dietary_fibers _Q4	0.476
LTAS	*A. muciniphila*	dietary_fibers	0.340	4487.136	4488.227	continuous	NA	*A. muciniphila*:dietary_fibers	0.335
	A. muciniphila	dietary_fibers_Q	0.321	4487.596	4490.102	categorical	Q1, Q2, Q3, Q4	*A. muciniphila*:dietary_fibers _Q2	0.069
								*A. muciniphila:*dietary_fibers _Q3	0.282
								*A. muciniphila:*dietary_fibers _Q4	0.207

Results of the Cox proportional hazards models evaluating the interaction between *Akkermansia muciniphila* (exposure) and dietary fiber intake (interaction variable) on the risk of T2D. The analysis compares models with and without the interaction term.
FAS: Full Analysis Set (n_{total}=4685 with 383 T2D incident cases**)**, LTAS: Lag Time Analysis Set (n_{total}=4,633 with 331 T2D incident cases**)**.
lrt p-value: P-value from the likelihood ratio test comparing the models with and without the interaction term.
aic_no_int: Akaike Information Criterion (AIC) for the model without the interaction term,
aic_with_in: AIC for the model including the interaction term.
Models were adjusted for all previous covariates i.e, sex, baseline age, level of education, height and waist, smoking status, walking/cycling, physical exercise, coffee consumption, daily intakes of total energy, alcohol, yogurt, red/processed meat, sugary food/sweetened beverages, statin medication use, aliquoting plate, and sequencing depth, except for wholegrains.

Table S4. Robust features association with incident T2D in the Full Analysis Set and the Lag Time Analysis Set adjusting for fasting plasma glucose. Related to Figures 1 and 4.

	**Full Analysis Set**			**Lag Time Analysis Set**		
**Species features**	HR 95% IC	p-value	q-value	HR	p-value	q-value
Alistipes_communis	1.053 (95% CI, 1.02-1.088)	**0.002**	**0.010**	1.048 (95% CI, 1.012-1.084)	**0.008**	**0.031**
Ruminococcus_gnavus	1.026 (95% CI, 1.004-1.048)	**0.021**	**0.045**	1.023 (95% CI, 0.999-1.047)	*0.061*	*0.081*
Desulfovibrio_piger	1.018 (95% CI, 0.993-1.044)	0.160	0.192	1.029 (95% CI, 1.001-1.056)	**0.039**	*0.081*
Akkermansia_muciniphila	1.018 (95% CI, 0.994-1.042)	0.149	0.192	1.026 (95% CI, 1-1.053)	**0.047**	*0.081*
Alistipes_finegoldii	1.011 (95% CI, 0.985-1.038)	0.407	0.444	1.012 (95% CI, 0.984-1.041)	0.416	0.416
GGB3614_SGB4886	1.01 (95% CI, 0.983-1.038)	0.475	0.475	1.016 (95% CI, 0.987-1.046)	0.283	0.308
Coprococcus_catus	0.976 (95% CI, 0.945-1.008)	0.137	0.192	0.975 (95% CI, 0.942-1.009)	0.153	0.183
Erysipelotrichaceae_bacterium	0.972 (95% CI, 0.949-0.996)	**0.023**	**0.045**	0.975 (95% CI, 0.95-1)	*0.054*	*0.081*
Clostridia_unclassified_SGB6317	0.953 (95% CI, 0.924-0.982)	**0.002**	**0.010**	0.958 (95% CI, 0.928-0.989)	**0.008**	**0.031**
**Gut Metabolic Modules (GMMs)**						
MF0042_asparagine degradation	1.088 (95% CI, 1.016-1.164)	**0.015**	**0.045**	1.092 (95% CI, 1.015-1.174)	**0.018**	*0.055*
MF0018_mannose degradation	0.589 (95% CI, 0.389-0.893)	**0.013**	**0.045**	0.524 (95% CI, 0.337-0.816)	**0.004**	**0.031**
MF0071 pentose phosphate pathway (non oxidative branch)	0.486 (95% CI, 0.19-1.243)	0.132	0.192	0.349 (95% CI, 0.126-0.964)	**0.042**	*0.081*

Cox regression models adjusted for sex, baseline age, level of education, height and waist circumference, smoking status, walking/cycling, physical exercise, coffee consumption, daily intakes of total energy, alcohol, wholegrains, yogurt, red/processed meat, sugary food/sweetened beverages, statin medication use, aliquoting plate, and sequencing depth and **fasting plasma glucose**. p-values and q-values < 0.05 are marked in bold and p-values and q-values < 0.1 are marked in italic.

Table S5. Subdistribution hazard ratios for incident type 2 diabetes from Fine and Gray competing risk models, considering death as a competing event. Related to Figures 1 and 4.

	Full Analysis Set			Lag Time Analysis Set		
**Species features**	SHR 95% IC	p-value	q-value	SHR 95% IC	p-value	q-value
Alistipes_communis	1.045 (95% CI, 1.013-1.078)	**0.005**	**0.008**	1.037 (95% CI, 1.004-1.071)	**0.028**	**0.033**
Ruminococcus_gnavus	1.035 (95% CI, 1.014-1.057)	**0.001**	**0.003**	1.032 (95% CI, 1.008-1.056)	**0.0083**	**0.015**
Desulfovibrio_piger	1.033 (95% CI, 1.010-1.058)	**0.006**	**0.008**	1.034 (95% CI, 1.008-1.061)	**0.009**	**0.015**
Akkermansia_muciniphila	1.027 (95% CI, 1.004-1.051)	**0.023**	**0.025**	1.032 (95% CI, 1.007-1.058)	**0.013**	**0.017**
Alistipes_finegoldii	1.034 (95% CI, 1.008-1.060)	**0.011**	**0.015**	1.027 (95% CI, 0.999-1.056)	*0.062*	*0.068*
GGB3614_SGB4886	1.023 (95% CI, 0.997-1.050)	*0.087*	*0.087*	1.025 (95% CI, 0.996-1.054)	*0.088*	*0.088*
Coprococcus_catus	0.961 (95% CI, 0.932-0.992)	**0.015**	**0.018**	0.957 (95% CI, 0.924-0.990)	**0.012**	**0.017**
Erysipelotrichaceae_bacterium	0.960 (95% CI, 0.938-0.984)	**0.001**	**0.003**	0.962 (95% CI, 0.937-0.987)	**0.0028**	**0.006**
Clostridia_unclassified_SGB6317	0.949 (95% CI, 0.923-0.976)	**0**	**0.001**	0.954 (95% CI, 0.927-0.982)	**0.0016**	**0.005**
**Gut Metabolic Modules (GMMs)**						
MF0042_asparagine degradation	1.124 (95% CI, 1.056-1.197)	**0**	**0.001**	1.119 (95% CI, 1.044-1.199)	**0.0015**	**0.004**
MF0018_mannose degradation	0.446 (95% CI, 0.304-0.653)	**0**	**0**	0.438 (95% CI, 0.291-0.660)	**0**	**0**
MF0071 pentose phosphate pathway (non oxidative branch)	0.256 (95% CI, 0.099-0.662)	**0.005**	**0.008**	0.188 (95% CI, 0.067-0.527)	**0.0015**	**0.005**

Subdistribution hazard ratios (SHRs) for incident type 2 diabetes estimated using Fine and Gray competing risk models, treating death as a competing event. Models were adjusted for sex, baseline age, level of education, height and waist circumference, smoking status, walking/cycling, physical exercise, coffee consumption, daily intakes of total energy, alcohol, wholegrains, yogurt, red/processed meat, sugary food/sweetened beverages, statin medication use, aliquoting plate, and sequencing depth.

Table S6. Complete cases analysis including 3114 participants without missing data for the covariates based on the Full Analysis Set. Related to Figures 1 and 4.

Diversity metrics	HR 95% CI	p-value	q-value
PC1	1.005 (95% CI, 1-1.01)	0.054	0.187
PC2	1.003 (95% CI, 0.995-1.011)	0.491	0.491
PC3	0.996 (95% CI, 0.986-1.006)	0.404	0.491
PC4	0.996 (95% CI, 0.986-1.007)	0.491	0.491
PC5	1.011 (95% CI, 0.999-1.022)	0.07	0.187
PC6	1.012 (95% CI, 0.999-1.024)	0.063	0.187
Shannon	0.785 (95% CI, 0.572-1.077)	0.134	0.214
Species richness	0.998 (95% CI, 0.996-1)	0.095	0.19
**Species features**			
Bilophila_wadsworthia	1.048 (95% CI, 1.009-1.089)	**0.015**	0.051
Akkermansia_muciniphila	1.046 (95% CI, 1.015-1.077)	**0.003**	**0.014**
Alistipes_communis	1.04 (95% CI, 1.002-1.079)	**0.038**	0.096
Desulfovibrio_piger	1.039 (95% CI, 1.009-1.069)	**0.010**	**0.039**
Ruminococcus_gnavus	1.029 (95% CI, 1.003-1.056)	**0.028**	0.081
Alistipes_finegoldii	1.029 (95% CI, 0.997-1.062)	0.077	0.127
Parabacteroides_distasonis	1.028 (95% CI, 0.979-1.078)	0.266	0.340
Alistipes_putredinis	1.026 (95% CI, 0.983-1.071)	0.233	0.320
GGB3614_SGB4886	1.02 (95% CI, 0.987-1.053)	0.237	0.320
GGB9712_SGB15244	1.007 (95% CI, 0.977-1.036)	0.663	0.727
Bacteroides_caccae	1.003 (95% CI, 0.972-1.034)	0.857	0.857
Dorea_longicatena	0.99 (95% CI, 0.936-1.048)	0.733	0.767
GGB4585_SGB6340	0.987 (95% CI, 0.959-1.016)	0.376	0.432
Coprococcus_eutactus	0.986 (95% CI, 0.96-1.014)	0.329	0.399
Bacteroides_faecis	0.981 (95% CI, 0.952-1.011)	0.214	0.320
Clostridia_unclassified_SGB14844	0.975 (95% CI, 0.949-1.003)	0.077	0.127
Blautia_sp_AF19_10LB	0.975 (95% CI, 0.947-1.003)	0.077	0.127
Ruminococcaceae_unclassified_SGB4191	0.972 (95% CI, 0.945-0.999)	**0.042**	0.096
Coprococcus_catus	0.965 (95% CI, 0.93-1.001)	0.054	0.112
Erysipelotrichaceae_bacterium	0.955 (95% CI, 0.928-0.982)	**0.001**	**0.008**
Bacilli_bacterium	0.953 (95% CI, 0.927-0.981)	**0.001**	**0.007**
Clostridia_unclassified_SGB6317	0.94 (95% CI, 0.908-0.973)	**0.001**	**0.006**
GGB3478_SGB4643	0.933 (95% CI, 0.902-0.965)	**0**	**0.001**
**Gut Metabolic Modules (GMMs)**			
MF0042_asparagine degradation	1.116 (95% CI, 1.029-1.21)	**0.008**	0.149
MF0018_mannose degradation	0.363 (95% CI, 0.215-0.612)	**0**	**0.015**
MF0071 pentose phosphate pathway (non oxidative branch)	0.239 (95% CI, 0.078-0.726)	**0.012**	0.149

Cox regression models adjusted for sex, baseline age, level of education, height and waist circumference, smoking status, walking/cycling, physical exercise, coffee consumption, daily intakes of total energy, alcohol, wholegrains, yogurt, red/processed meat, sugary food/sweetened beverages, statin medication use, aliquoting plate, and sequencing depth. p-values and q-values < 0.05 are marked in bold.
